# Gut Microbiota‐Derived Extracellular Vesicles in Patients With Obesity Undergoing Gastric Bypass Surgery

**DOI:** 10.1111/mmi.70064

**Published:** 2026-03-12

**Authors:** Jenni Hekkala, Anna Kaisanlahti, Mysore V. Tejesvi, Jenni Turunen, Nikke Virtanen, Sonja Karikka, Pande Putu Erawijantari, Anatoliy Samoylenko, Genevieve Bart, Seppo Vainio, Leo Lahti, Janne Hukkanen, Terhi Ruuska‐Loewald, Vesa Koivukangas, Justus Reunanen

**Affiliations:** ^1^ Research Unit of Translational Medicine University of Oulu Oulu Finland; ^2^ Biocenter Oulu University of Oulu Oulu Finland; ^3^ Research Unit of Clinical Medicine University of Oulu Oulu Finland; ^4^ Laboratory of Developmental Biology, Disease Networks Research Unit, Faculty of Biochemistry and Molecular Medicine University of Oulu Oulu Finland; ^5^ Department of Computing University of Turku Turku Finland; ^6^ Kvantum Institute University of Oulu Oulu Finland; ^7^ Research Unit of Biomedicine and Internal Medicine, Medical Research Center Oulu Oulu University Hospital and University of Oulu Oulu Finland; ^8^ Department of Pediatrics and Adolescent Medicine Oulu University Hospital Oulu Finland

**Keywords:** extracellular vesicles, gastric bypass surgery, gut microbiota, nanoparticles, obesity

## Abstract

Human gut microbiota is associated with obesity. Gut microbiota–derived extracellular vesicles (EVs), lipid coated nanoparticles secreted by bacteria, have been suggested as a communication mechanism between gut microbiota and the host. This study characterized the effect of Roux‐en‐Y gastric bypass (RYGB) on gut microbiota and gut microbiota–derived EVs in patients with obesity. Fecal samples were collected from 30 recruited patients at baseline and 6 months after surgery. EVs were isolated from fecal samples, and their origin and protein content were analyzed. The number of unique proteins was increased in gut microbiota–derived EVs after the surgery as compared to baseline. A significant difference in both microbiota composition (*p* = 0.001; PERMANOVA) and microbiota–derived EVs (*p* = 0.001; PERMANOVA) was observed in response to surgery. Based on 16S rRNA gene sequencing data, a random forest classifier accurately classified both gut microbiota (AUC = 0.93) and EVs (AUC = 0.80) to baseline and after surgery groups. This study found that gastric bypass surgery altered both the composition and characteristics of gut microbiota and gut microbiota–derived EVs in patients with obesity. Thus, gut microbiota–derived EVs may play a role in obesity and influence the health effects of bariatric surgery beyond the gut.

**Trial Registration:** ClinicalTrials.gov identifier: NCT00950003

## Graphical abstract

Extracellular vesicles (EVs) have been suggested to participate in gut microbiota–host communication. While obesity and bariatric surgery have been shown to alter the gut microbiota composition, changes in gut microbiota‐EVs have not been investigated. We show that gastric bypass surgery affected the protein cargo and composition of gut microbiota–derived EVs distinctly from changes in gut microbiota composition. Gut microbiota–derived EVs may play a role in obesity and health effects of bariatric surgery.

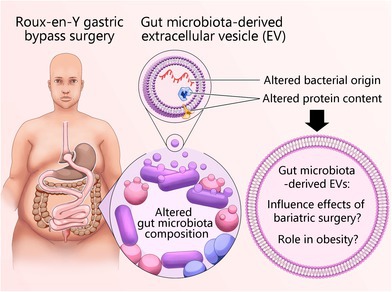

## 1 Introduction

Obesity is a multifactorial disease driven by a complex interplay of genetic, cultural, and societal factors, and it remains a primary risk factor for chronic metabolic conditions (Weinsier et al. 1998; Milhem and Komarnytsky 2023). Roux‐en‐Y gastric bypass (RYGB) is one of the most effective treatments for severe obesity (Arterburn et al. 2020; Mingrone et al. 2021). While RYGB physically restricts meal sizes and alters satiety regulation, it also results in profound physiological changes, including changes in the gut environment.

The gut microbiota is a central regulator of host health, playing essential roles in immune system maturation, intestinal barrier integrity, and nutrient balance (Brown et al. 2019; Villard et al. 2021). Alteration in the microbial composition has been strongly linked with obesity and comorbidities such as type 2 diabetes (T2D) (Ley et al. 2006; Turnbaugh et al. 2008, 2009; Ruuskanen et al. 2022).

RYGB triggers a rapid and distinct shift in the gut microbiota (Walker et al. 2010; Tremaroli et al. 2015; Palleja et al. 2016; Murphy et al. 2017; Koffert et al. 2020), typically within three months after surgery (Furet et al. 2010; Dang et al. 2022). While these microbial alterations likely have long‐term effects on the host's metabolism (Tremaroli et al. 2015), the exact mechanisms by which gut bacteria communicate with peripheral tissues remain poorly understood. One emerging pathway for this inter‐kingdom communication is the secretion of gut microbiota–derived extracellular vesicles (EVs). These lipid–bilayered nanoparticles carry a diverse cargo of proteins, metabolites, lipids, and nucleic acids (Kim et al. 2015). Unlike the bacteria themselves, microbiota–derived EVs can cross the intestinal barrier and enter the bloodstream, potentially extending the influence of gut microbiota beyond from gastrointestinal tract to distant organs (Choi et al. 2015; Park et al. 2017; Nah et al. 2019). There is a significant knowledge gap regarding how these vesicles behave in the context of surgical weight loss. Despite the well documented changes in gut microbiota composition after RYGB (Walker et al. 2010; Furet et al. 2010; Tremaroli et al. 2015; Palleja et al. 2016; Murphy et al. 2017; Koffert et al. 2020; Dang et al. 2022), data on how these changes translate to the EVs secreted by gut microbiota, and the proteomic cargo within EVs, are largely missing.

In this study, we investigated the longitudinal characteristics of gut microbiota–derived EVs alongside the gut microbiota in patients with obesity at baseline and 6 months after RYGB. By characterizing both the bacterial 16S rRNA to assess EV origin and the EV proteome, we aimed to elucidate how RYGB changes the systemic signaling of the gut microbiota.

## 2 Results

### 2.1 Study Population

**FIGURE 1:**
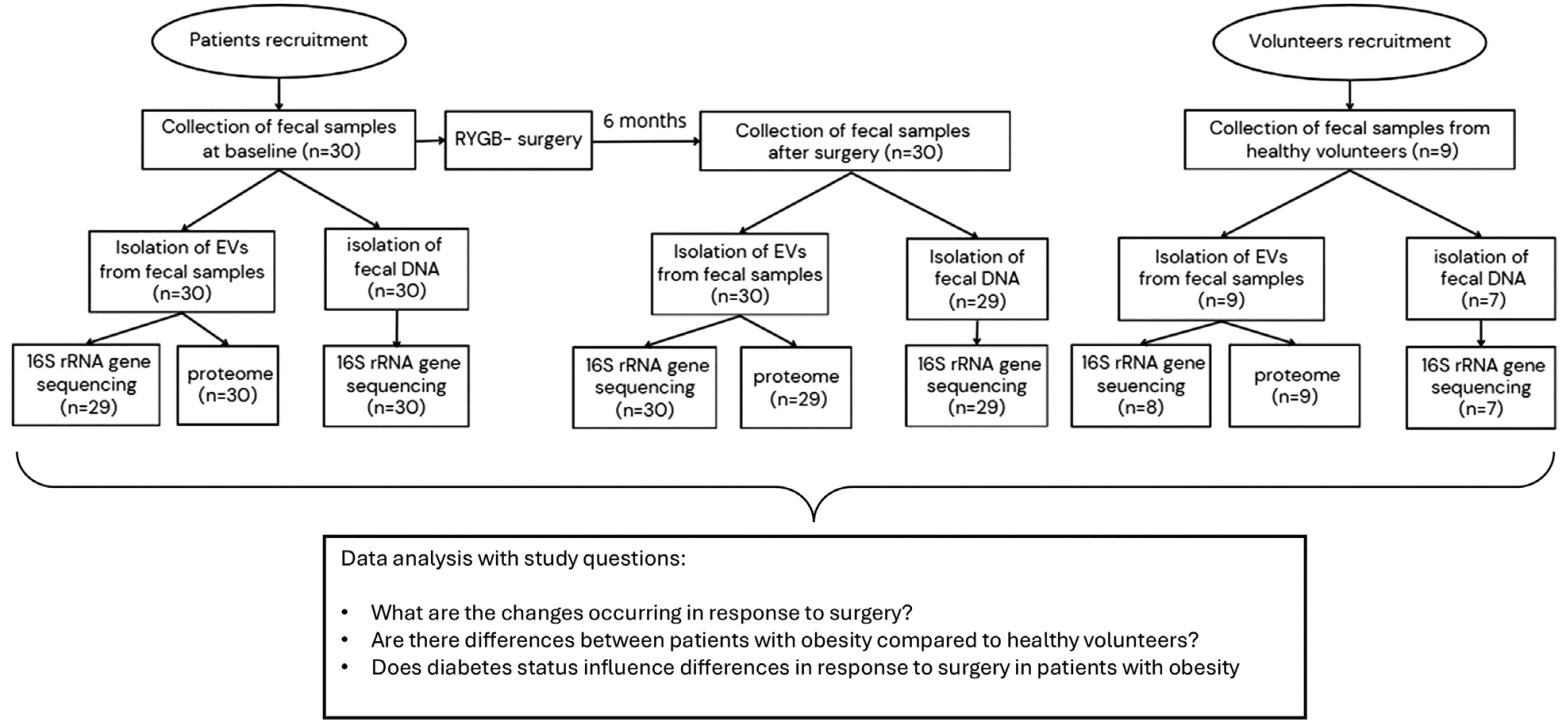
Study design.

We collected fecal samples from 30 patients undergoing gastric bypass surgery. The study design is described in Figure 1. From patients with obesity, 70% were females, with a mean age of 47.3 years and a mean BMI of 44.5. On average, they lost 27.2 kg of body weight within 6 months after surgery (Table S1). Recruited patients were also divided into subpopulations if they had T2D as a comorbidity (*n* = 16) or without (*n* = 14). In addition, fecal samples were collected from healthy volunteers (*n* = 9) with a mean age of 46.3 years and a mean BMI of 26.1 (Table S1).

### 2.2 Characterization of Extracellular Vesicles

After isolating EVs, we confirmed their presence using transmission electron microscopy (TEM) imaging (Figure S1). We imaged fractions 6 and 7 from the density gradient, confirming the purity of the EV solution by the minimal presence or complete absence of cellular debris, flagella, or fibers.

In nanoparticle tracking analysis (NTA), we defined the size distribution and concentration of the EVs (Table 1). In the subgroup of patients with obesity and T2D, the mean diameter of EVs was statistically smaller after surgery (mean 210 [SD 76] nm vs. 175 [SD 70] nm, *p* = 0.048).

**TABLE 1:** Size distribution (nm) with standard deviation (SD) and concentration (particles/ml) with standard deviation (SD) of EV in study groups.

	Size distribution (nm)				Concentration (particles/ml)			
	At baseline		After surgery		At baseline		After surgery	
	Mean	SD	Mean	SD	Mean	SD	Mean	SD
ALL OB	190	56	164	27	1.1 × 10^8^ *	1.5 × 10^8^	4.6 × 10^8^ *	2.9 × 10^8^
Subgroup of OB w/o T2D	182	69	158	59	1.2 × 10^8^ *	6.0 × 10^7^	4.8 × 10^8^ *	3.0 × 10^8^
Subgroup of OB w T2D	210*	76	175*	70	1.7 × 10^8^ *	2.0 × 10^8^	6.0 × 10^8^ *	2.9 × 10^8^
Healthy volunteers	271	106			3 × 10^8^	1.6 × 10^8^		

* Statistically significant difference according to *t*‐test or paired *t*‐test (*p* < 0.05).

The mean concentration of EVs increased significantly after surgery (mean 1.1 × 10^8^ [SD 1.5 × 10^8^] particles/ml vs. mean 4.6 × 10^8^ [SD 2.9 × 10^8^] particles/ml, *p* < 0.001) in all patients with obesity. We observed a significant increase after surgery in both the subgroup with obesity and without T2D (mean 1.2 × 10^8^ [SD 6.0 × 10^7^] particles/ml vs. mean 4.8 × 10^8^ [SD 3.0 × 10^8^] particles/ml, *p* < 0.001) and the subgroup of patients with obesity and T2D (mean 1.7 × 10^8^ [SD 2.0 × 10^8^] particles/ml vs. mean 6.0 × 10^8^ [SD 2.9 × 10^8^] particles/ml, *p* < 0.001).

### 2.3 Proteome

The proteome sequences of isolated EVs identified from the UniProtKB/Swiss‐Prot resulted in 3634 recognized peptides corresponding to 1426 protein IDs, whereas those identified from UniProtKB/trEMBL resulted in 23,350 recognized peptides and 18,369 protein IDs. Overall, 995 human proteins and 11,436 bacterial proteins were identified. In the subgroup of patients with obesity and without T2D (*n* = 14), the mean number of identified bacterial proteins was 1460 (range 84–4241) per sample at baseline; after the surgery (*n* = 14), the mean number of identified proteins was 2324 (range 229–4230) per sample. In the subgroup of patients with obesity and T2D (*n* = 16), the mean number of identified bacterial proteins per sample was, at baseline, 907 (range 8–3000) and after the surgery (*n* = 15), it was 2964 (range 256–4970). The mean number of identified bacterial proteins per sample was 2982 (range 1256–5016) in the group of volunteers (*n* = 9).

**FIGURE 2:**
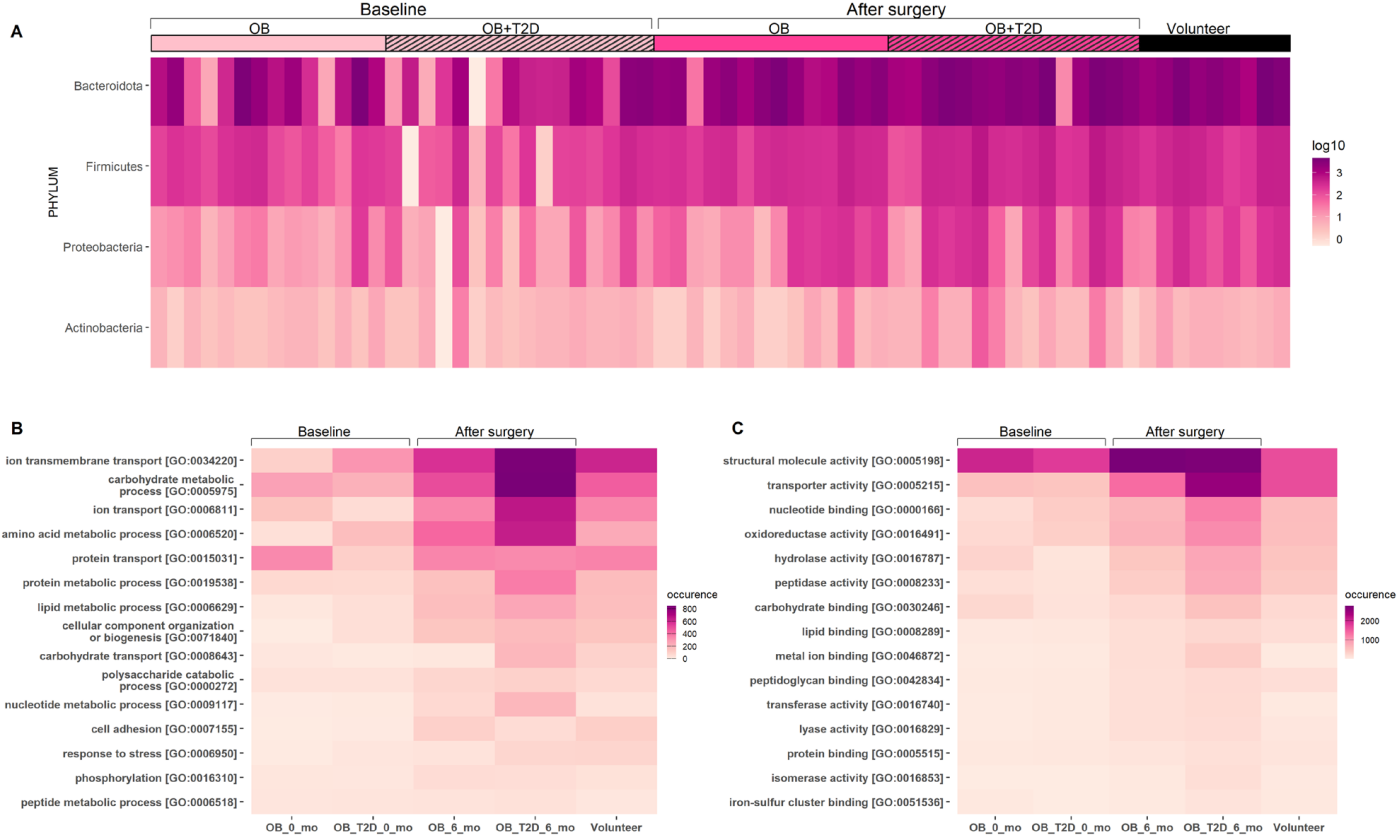
(A) Presence of unique gut microbiota–derived EV proteins based on origin in four phyla in log10 scale in the study groups; (B) the 15 most prevalent GO annotation classes of bacterial proteins based on biological process; (C) molecular functions. At baseline: OB/OB_0_mo = patients with obesity without T2D at baseline (*n* = 14); OB + T2D/OB_T2D_0_mo = patients with obesity and T2D at baseline (*n* = 16). After surgery: OB/OB_6_mo = patients with obesity without T2D 6 months after surgery (*n* = 14); OB + T2D/OB_T2D_6_mo = patients with obesity and T2D 6 months after surgery (*n* = 15), Volunteer (*n* = 9).

Overall, 58% of bacterial EV proteins were shared between the healthy volunteers control group and all patients with obesity at baseline and after the surgery (Figure S2). Altogether, 3% of the bacterial proteins were present only in all patients with obesity at baseline, and 16% after the surgery. In all patients undergoing RYGB, 9% of the identified bacterial proteins were shared at baseline and after surgery. Overall, 2% of the identified bacterial EV proteins were present only in the group of healthy volunteers. The predominant bacterial EV proteins were recognized to originate from the Bacteroidota phylum in all the study groups, followed by those originating from Firmicutes, Proteobacteria, and Actinobacteria (Figure 2A).

To explore the functional landscape of the identified proteins, we performed Gene Ontology (GO) mapping. Altogether, 6%–11% of the identified bacterial proteins had GO annotation in biological process class. Regarding gene ontology for biological processes, the most prevalent terms shared by all groups were transmembrane transport, carbohydrate metabolic process, ion transport, amino acid metabolic process, and protein transport (Figure 2B). In molecular function class, 18%–21% of bacterial EV proteins identified had GO annotation available. For molecular function, the predominant terms were structural molecule activity, transporter activity, nucleotide binding, oxidoreductase activity, and hydrolase activity (Figure 2C).

A longitudinal comparison revealed a marked expansion in the diversity of the identified EV proteome following RYGB. Six months after surgery, we observed a prominent increase in the proteins associated with key metabolic and structural pathways. Specifically, there was enrichment of proteins involved in ion transmembrane transport, carbohydrate metabolic processes, ion transport and amino acid metabolic processes (Figure 2B). In the class of molecular functions, the diversity of proteins related to structural molecule activity and transporter activity increased compared to baseline (Figure 2C).

### 2.4 Bacteriome Analysis

The taxonomic composition of gut microbiota in feces (FE) and the bacterial origin of EVs were analyzed using 16S rRNA gene sequencing. After quality control, the remaining sample numbers were as follows: patients with obesity and without T2D at baseline (FE: *n* = 14, EV: *n* = 14) and after the surgery (FE: *n* = 13, EV: *n* = 14), patients with obesity and T2D at baseline (FE: *n* = 16, EV: *n* = 15) and after the surgery (FE: *n* = 16, EV: *n* = 16), and in healthy volunteers group (FE: *n* = 8, EV: *n* = 9).

**FIGURE 3:**
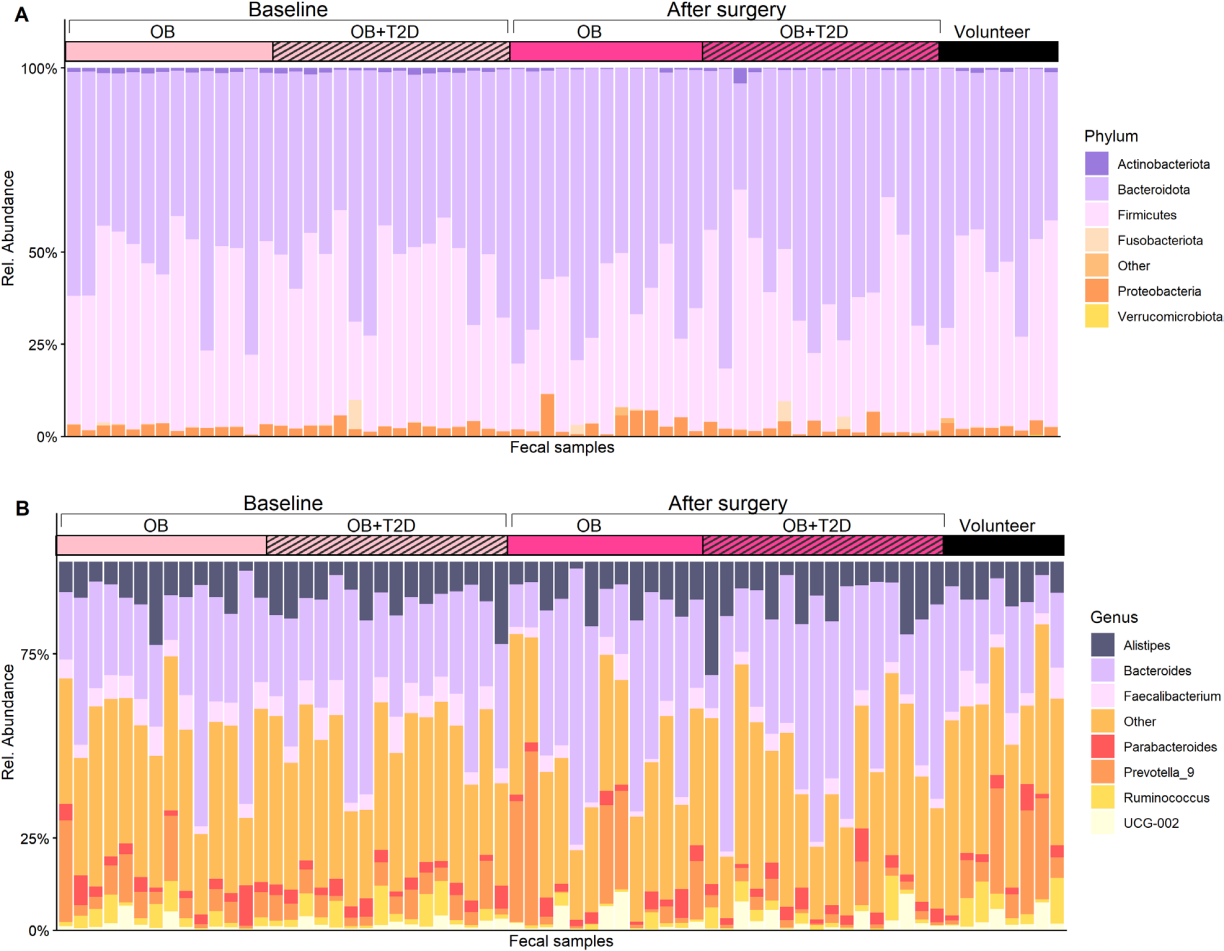
The 7 most abundant phyla and genera by relative abundance of (A) fecal 16 s rRNA at the phylum level and (B) fecal 16 s rRNA at the genus level. At baseline, OB = patients with obesity at baseline (*n* = 14); OB + T2D = patients with obesity and T2D at baseline (*n* = 16). After Surgery: OB = patients with obesity 6 months after surgery (*n* = 13); OB + T2D = patients with obesity and T2D 6 months after surgery (*n* = 16); Volunteer = group of healthy volunteers (*n* = 8).

The relative abundance in the samples was visualized individually (Figure 3), and by study group (Figure S3). The most abundant phylum in the gut microbiota of all the study groups was Bacteroidota, followed by Firmicutes, Proteobacteria, Actinobacteria, and Fusobacteria (Figure 3 and Table S2). The most abundant genus in the gut microbiota of all groups was *Bacteroidetes*, followed by *Prevotella_9*, *Alistipes, Faecalibacterium, Ruminococcus*, and *Parabacteroidetes* (Table S3).

**FIGURE 4:**
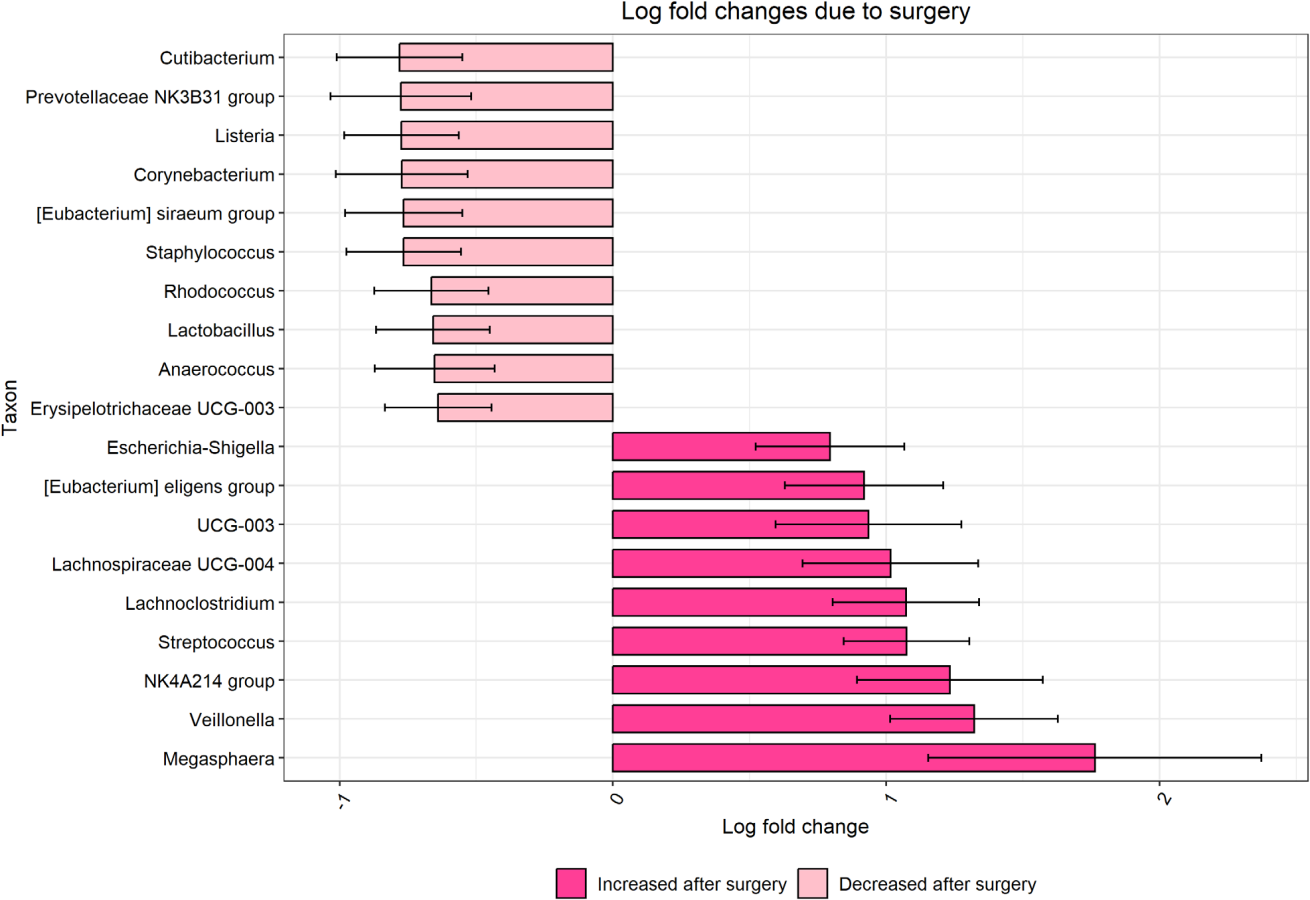
Log fold change of statistically significant (*q* < 0.05) genera in ANCOM‐BC analysis within paired samples and FRD correction. Differential abundance of gut microbiota in patients with obesity at baseline and after surgery.

According to differential abundance analysis, Analysis of Composition of Microbiomes with Bias Correction (ANCOM‐BC), at the phylum level, in gut microbiota, the abundance of Actinobacteriota decreased and Verrucomicrobiota increased after gastric bypass surgery in all patients with obesity (*q* < 0.05, ANCOM‐BC, Table S4). At the genus level, a significant difference was observed in abundance of 19 bacterial genera, with an increasing abundance of *Megasphaera, Veillonella, Streptococcus* and *Lachnoclostridium*, and a decreasing abundance of *Cutibacterium*, *Prevotellaceae, Listeria, Corynebacterium, Eubacterium siraeum* group and *Staphylococcus* (ANCOM‐BC, Figure 4 and Table S5).

In differential abundance analysis of gut microbiota comparing at baseline and after the surgery in the subgroups of patients with obesity and without T2D, revealed an increase in Bacteroidota and a decrease in Actinobacteriota, from which the decrease of Actinobacteriota was statistically significant (*q* < 0.05, Table S6). At genus level there was no significant change. In the subgroup of patients with obesity and T2D, the abundance of Bacteroidota and Firmicutes increased after the surgery whereas Actinobacteriota decreased, but not significantly (*q* > 0.05, Table S2). At the genus level, there was no significant change. A comparison of the healthy volunteers group with all patients with obesity at baseline revealed significantly higher abundance in one genus *Allorhizobium‐Neorhizobium‐Pararhizobium‐Rhizobium* in healthy volunteers (ANCOM‐BC, Table S7). A comparison of the healthy volunteers group with all patients with obesity after surgery revealed significantly higher abundance in three genera *Veillonella, Lachnoclostridium* and *Streptococcus* after surgery (ANCOM‐BC, Table S8).

**FIGURE 5:**
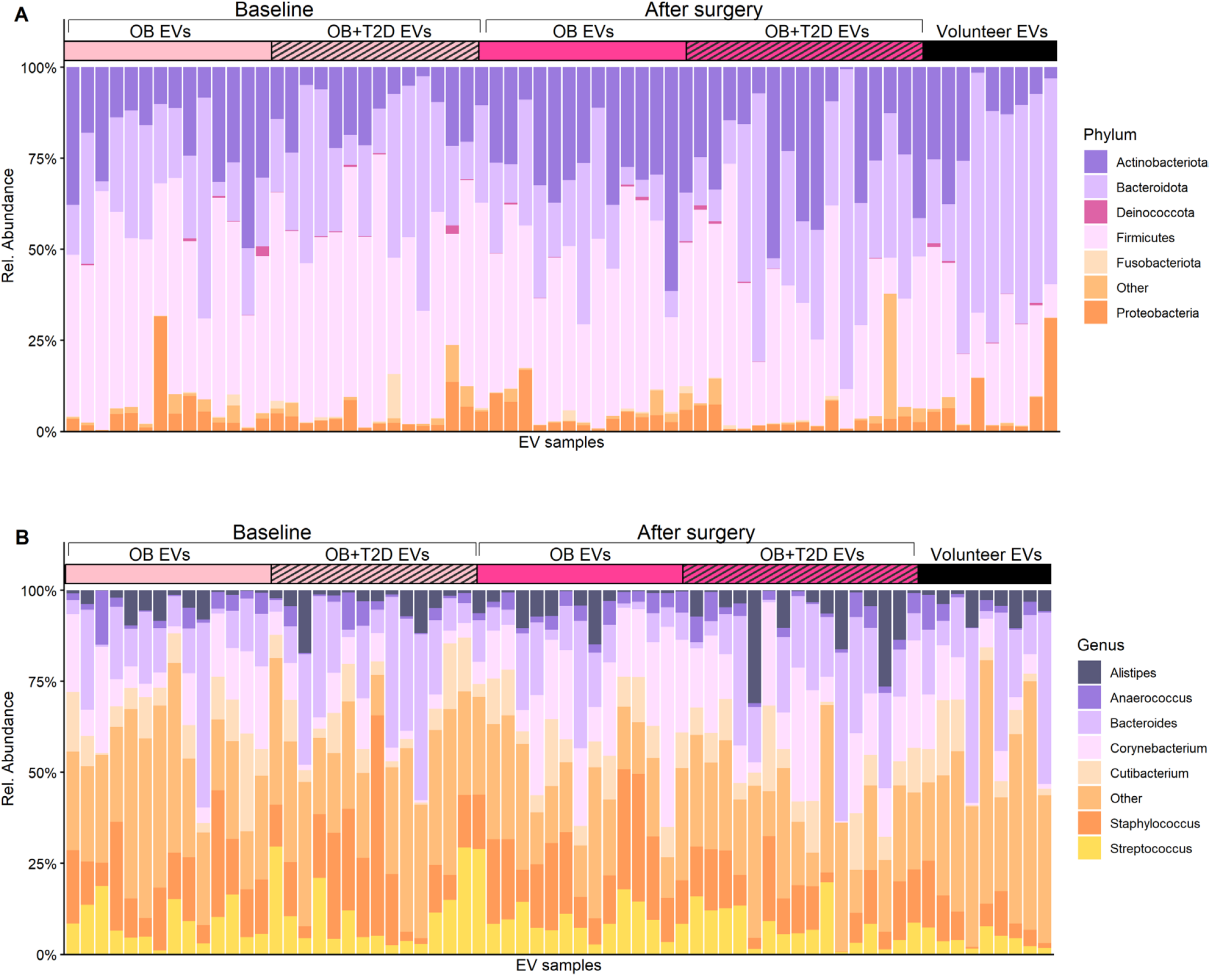
The 6 most abundant phyla and genera by relative abundance of (A) 16 s rRNA of gut microbiota–derived EVs at the phylum level. (B) 16 s rRNA of gut microbiota–derived EVs at the genus level. At Baseline: OB EVs = patients with obesity at baseline (*n* = 14); OB + T2D EVs = patients with obesity and T2D at baseline (*n* = 15). After surgery: OB EVs = patients with obesity 6 months after surgery (*n* = 14); OB + T2D EVs = patients with obesity and T2D 6 months after surgery (*n* = 16), Volunteer = healthy volunteer group (*n* = 9).

The most abundant phylum in the EV samples was Bacteroidota in the healthy volunteers group and in the subgroup of patients with obesity and T2D after surgery (Figure 5A and Table S2). In the other groups, the most abundant phylum was Firmicutes, followed by Bacteroidota/Firmicutes, Proteobacteria, Actinobacteriota, Deinococcota, and Fusobacteriota. In gut microbiota–derived EVs, the most abundant genera were *Bacteroides, Corynebacterium, Staphylococcus, Parabacteroides, Streptococcus, Cutibacterium, and Alistipes* (Figure 5B and Table S3).

Regarding the origin of EVs, comparing all patients with obesity at baseline and after surgery, there were no significant changes in genus‐level neither in subgroup analysis at baseline and after surgery. The healthy volunteers group had a significantly higher abundance of the genus *Prevotella_9* (ANCOM‐BC, Table S9) than all the patients with obesity at baseline in EVs. Healthy volunteers had a significantly higher abundance of the genera *Prevotella_9* and *Lactococcus* (ANCOM‐BC, Table S10) than all the patients with obesity after surgery in EVs.

**FIGURE 6:**
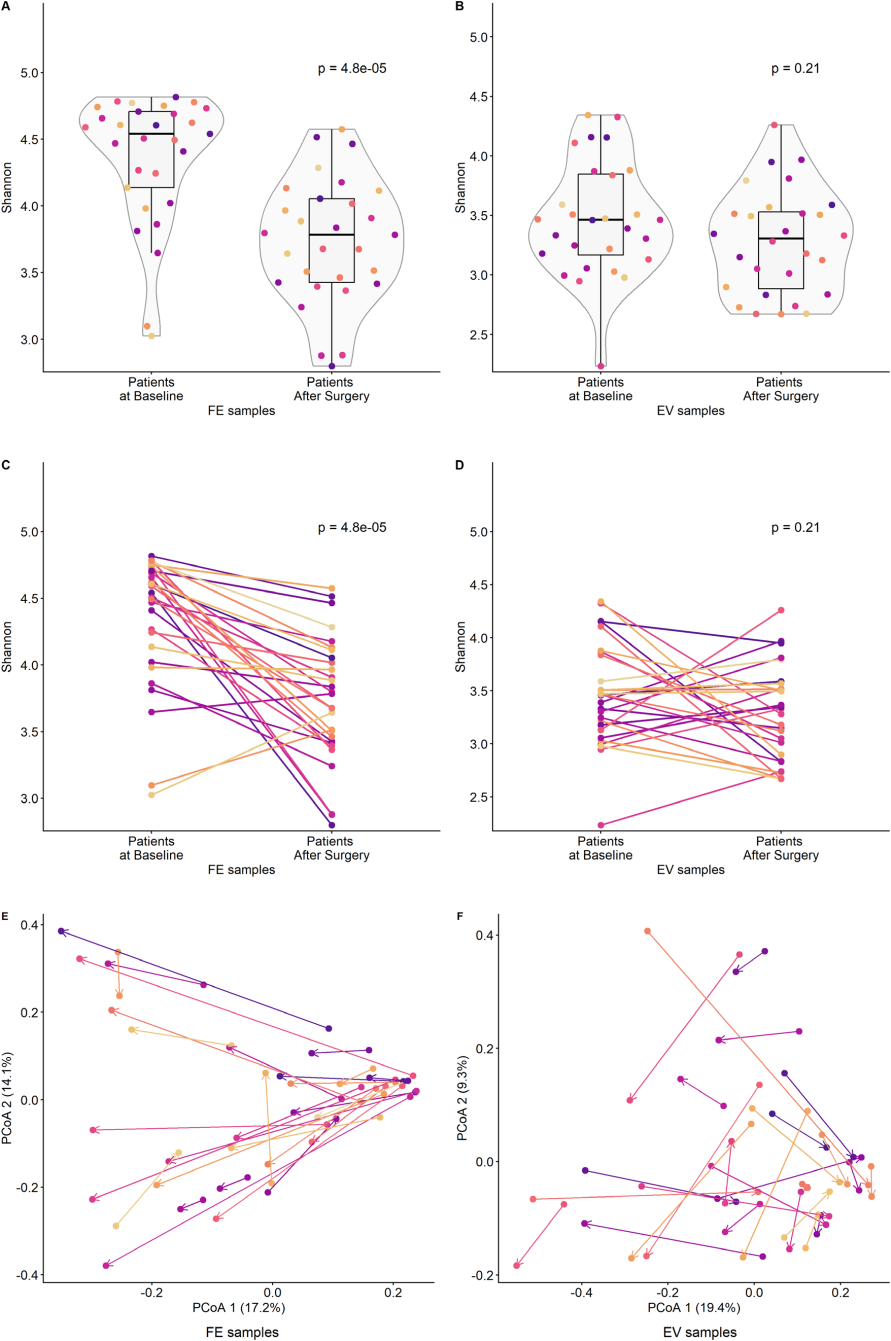
(A) *Alpha* diversity violin plot of gut microbiota by Shannon index, statistical significance by Wilcoxon signed rank with FDR correction. (B) *Alpha* diversity violin plot of gut microbiota–derived EVs by Shannon index, statistical significance by Wilcoxon signed rank with FDR correction. (C) *Alpha* diversity with subject‐level trajectories of gut microbiota. (D) *Alpha* diversity with subject‐level trajectories of gut microbiota derived EVs. (E) PCoA plot of gut microbiota with subject‐level trajectories performed using Bray–Curtis dissimilarity matrix, statistical significance by paired PERMANOVA (Effect size *R* ^2^ = 0.6035, *p* = 0.001). (F) PCoA plot of gut microbiota–derived EVs with subject‐level trajectories performed using Bray–Curtis dissimilarity matrix, statistical significance by paired PERMANOVA (Effect size *R* ^2^ = 0.5561, *p* = 0.002). Paired samples FE: *n* = 29, EV: *n* = 28.

In alpha diversity, measuring the diversity of a microbial community, the surgery induced a statistically significant decrease in the diversity of gut microbiota estimated by Shannon index with Wilcoxon signed rank (*p* < 0.001, Figure 6A,C). In beta diversity, which measures community similarities in gut microbiota, presented a significant difference between all patients with obesity at baseline and after surgery when estimated with Bray–Curtis dissimilarity (PERMANOVA: Effect size *R* ^2^ = 0.6035, *p* = 0.001, Figure 6E). In gut microbiota–derived EVs, the change in diversity induced by surgery was not significant, and there was no significant difference between all patients with obesity and the healthy volunteers (Figure 6B,D). In beta diversity, however, presented a significant difference in gut microbiota–derived EVs between all patients with obesity at baseline and after surgery (PERMANOVA: Effect size *R* ^2^ = 0.5561, *p* = 0.002) (Figure 6F).

While comparing to healthy volunteers, alpha diversity presented a statistically significant difference to all patients with obesity after surgery estimated by Shannon index with Wilcoxon rank sum with FRD (*p* < 0.01 Figure S4A). In beta diversity, a significant difference was observed between the volunteers group and all patients with obesity at baseline and after surgery in all comparisons when estimated with Bray–Curtis dissimilarity (PERMANOVA: Effect size, *R* ^2^ = 0.09255, *p* = 0.001, Figure S4B). In gut microbiota–derived EVs, there was no significant difference between all patients with obesity and the healthy volunteers (Figure S4C). In beta diversity, however, presented a significant difference in gut microbiota–derived EVs between the volunteers group and all patients with obesity at baseline and after surgery in all comparisons (PERMANOVA: Effect size *R* ^2^ = 0.06002, *p* = 0.002) (Figure S4D).

### 2.5 Machine Learning Classification of Fecal Samples and Fecal EV Samples Based on 16S Sequencing Results

**FIGURE 7:**
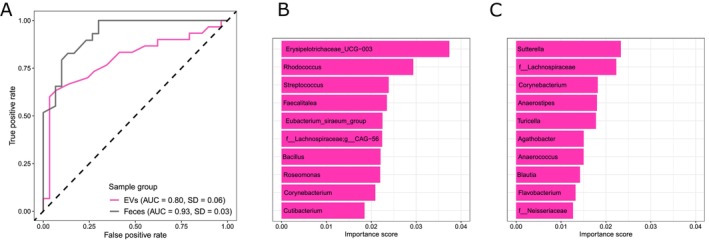
Sample classification of RYGB surgery patients at baseline and 6 months after surgery. (A) Receiver operating characteristics (ROCs) depicting the performance of the model built using the Random Forest algorithm. The true positive rate is in y‐axis, and the false positive rate is in x‐axis. The dashed line represents random chance. Area under curve (AUC) and standard deviation (SD) for EV and fecal sample groups have been included in the legend. (B) The 10 most important genera the model used to classify samples in the feces group in the order of importance. (C) The 10 most important genera the model used to classify samples in the EV group in the order of importance. 0 months = all patients with obesity at baseline (*n* = 30); 6 months = all patients with obesity 6 months after surgery (*n* = 30).

Samples from gastric bypass surgery patients were classified to belong either to baseline group (0 month) or after surgery group (6 months) using Random Forest algorithm to train a sample classifier. The classifier was accurate with both fecal samples (AUC = 0.93) and isolated fecal EV samples (AUC = 0.80) (Figure 7A) to those obtained at baseline and those obtained after surgery. The most important genera for the classification algorithm differed between fecal samples (gut microbiota) and EV samples (microbial origin of EVs). The most important genera for sample classification in the fecal samples group include UCG‐003 from the family *Erysipelotrichaceae*, as well as *Rhodococcus*, *Streptococcus*, and others (Figure 7B). In the EV group, the most important genera included *Sutterella*, as well as an unknown genus from the family *Lachnospiraceae*, *Corynebacterium*, and others (Figure 7C).

## 3 Discussion

This controlled study characterized the effect of gastric bypass surgery on gut microbiota and gut microbiota–derived EVs in patients with obesity. Our results show that gastric bypass decreased gut microbiota diversity and altered the composition of the microbial community. The observed changes in gut microbiota in response to surgery differed from the observed changes in the microbial origins of EVs, with the variety of EV proteomic cargo increasing in response to the surgery. The observed changes in gut microbiota–derived EVs suggest that the gut microbial effects of gastric bypass surgery may extend beyond the gut while the EVs are able to translocate to the circulation.

Our findings suggest that gastric bypass surgery may influence signaling between gut microbiota and the host by changing the composition of microbiota–derived EVs. Previously, obesity and comorbidities have been connected to the changes in gut microbiota composition (Turnbaugh et al. 2008; Gurung et al. 2020; Patra et al. 2023). In addition, several studies have reported alterations in gut microbiota after RYGB surgery (Tremaroli et al. 2015; Palleja et al. 2016; Murphy et al. 2017; Dang et al. 2022). Before the present study, however, there have been very limited data available on the effect of obesity or gastric bypass surgery on gut microbiota–derived EVs (Huh et al. 2019; Rodríguez‐Díaz et al. 2023). Previously, Huh et al. investigated the effect of RYGB on the composition of gut microbiota and gut microbiota–derived EVs in a rat model and observed noticeable surgery–induced changes in both (Huh et al. 2019). Microbial EVs have been previously reported to be able to enter the bloodstream (Park et al. 2017), and microbial EVs from serum have been described as having a similar relative abundance as gut microbiota in patients with T2D (Nah et al. 2019). Also in T2D, gut microbiota–derived EVs have been reported to be associated with the development of impaired glucose metabolism (Choi et al. 2015). The EVs of *Akkermansia municiphila* have been reported to exert beneficial health effects for the gut and against obesity in mouse models (Chelakkot et al. 2018; Ashrafian et al. 2019, 2021).

This study's analysis of EV proteomic cargo revealed an increased variety of unique bacterial proteins from baseline to after surgery. The most pronounced increase was observed in proteins related to the transport of ions and macronutrients, which may indicate a higher bacterial nutrient uptake caused by greater nutrient availability or increased efficiency for nutrient uptake for gut microbiota due to surgery. Previously, it has been reported that protein profiles in the gut microbiota of patients with obesity before and after bariatric surgery differ significantly (Sanchez‐Carrillo et al. 2021). More precisely, both the diversity and function of gut microbiota proteins have been reported to increase after surgery (Sanchez‐Carrillo et al. 2021). Our results demonstrate that the same effect appears to occur in gut microbiota–derived EVs, and the same phenomenon has been previously described in an animal model (Ferreira et al. 2022). Similarly, after gastric bypass surgery, the abundance of EV proteins from Bacteroidetes and Proteobacteria increased, which has previously been reported in the gut microbiota proteome in response to bariatric surgery (Sanchez‐Carrillo et al. 2021).

Both increase and decrease in gut microbiota diversity have been reported in response to RYGB (Palleja et al. 2016; Murphy et al. 2017; Crommen et al. 2020; Coimbra et al. 2022). Our results regarding the surgically–induced effect on gut microbiota are in line with these previous studies reporting a decrease in the diversity of gut microbiota composition (Palmisano et al. 2020; Dang et al. 2022) and an increase in the abundance of the phyla Bacteroidota and Proteobacteria (Coimbra et al. 2022). The abundance of Firmicutes remained roughly the same at baseline and after surgery as previously reported (Karami et al. 2021), but a change in genus‐level composition was observed, contradicting a previous report of a decrease in Firmicutes (Izhak et al. 2021; Özdemir et al. 2023). We observed a significant increase in aerotolerant genera, including *Veillonella*, *Streptococcus*, *Escherichia*‐*Shigella*, and members of the *Clostridia* class. A significant increase was observed in the abundance of Verrucomicrobiota as previously reported (Shen et al. 2019; Palmisano et al. 2020). In origin of EVs, we didn't observe any significant change in abundance at baseline and after surgery despite the change in gut microbiota composition. Still, our machine learning results support the phenomenon of significantly altered gut microbiota and microbiota–derived EVs in response to gastric bypass surgery.

Multiple studies have identified decreased microbial diversity in individuals with obesity compared to lean individuals (Ley et al. 2006; Turnbaugh et al. 2009; Bruce‐Keller et al. 2015). In our study, the genera *Prevotella_9* was differently abundant in gut microbiota–derived EVs when comparing the healthy volunteers' group to all patients with obesity at baseline. Previously Rodríguez‐Díaz et al. (2023) compared the taxonomic composition of gut microbiota and gut microbiota–derived EVs in a few different clinical conditions, including nine patients with morbid obesity. They didn't observe lower abundance in EVs originating from *Prevotella_9* or *Lactococcus*, which both had lower abundance when comparing healthy volunteers to patients after surgery. The difference in abundance was not observed in gut microbiota, although a high abundance of *Prevotella* has been connected to lower body weight in previous research (Christensen et al. 2019). In the present study, *Veillonella* and *Streptococcus* were significantly more abundant in the gut microbiota of all patients after surgery as compared to the healthy volunteers' group. An increased abundance of this oral–associated genera has been a common occurrence after gastric bypass surgery due to increased luminal pH and oxygen levels (Duncan et al. 2009; Stemmer et al. 2013).

The main strength of this study is the longitudinal characterization of gut microbiota–derived EVs, key mediators in inter‐kingdom signaling, alongside the shifts in the gut microbiota following gastric bypass surgery. The study design allowed for paired statistical analysis of samples before and after RYGB, supplemented with a group of healthy volunteers for cross‐sectional comparison. Furthermore, we employed a robust methodological framework for isolating and characterizing microbial EVs. The isolation protocol was specifically optimized to enrich microbial EVs, aligning with the primary focus of our research. To address the risk of contamination associated with the low biomass of biomolecules in EVs, we implemented a comprehensive set of extraction blanks, negative and positive controls along with computational filtering tools to identify and remove potential environmental contaminants to ensure the reliability of results.

This study also has certain limitations. The study cohort was relatively small, although the sample size was sufficient to detect significant longitudinal differences after RYGB. The number of participants in group heathy volunteers was relatively small and mean BMI of 26 kg/m^2^. Isolated EV pool likely represents a heterogenous population comprising both microbiome–derived EVs and host–derived vesicles. Regarding proteomic analysis, our data reflect the diversity of the proteome (i.e., the number of unique proteins identified) rather than absolute protein quantification. Furthermore, the functional analysis of the identified proteins was constrained by the limited availability of Gene Ontology (GO) annotations for many bacterial proteins. Finally, while we utilized comprehensive negative controls and bioinformatic tools to address potential environmental RNA contamination, this remains a persistent challenge inherent to the study of low‐biomass samples. Notably, even the application of advanced machine learning models cannot fully resolve the fundamental noise introduced by such contamination.

In conclusion, our results reveal alterations in both gut microbiota and gut microbiota–derived EVs in response to gastric bypass surgery. In addition to gut microbiota, gut microbiota–derived EVs may play a role in obesity and the health effects of bariatric surgery.

## 4 Experimental Procedures

We recruited the participants of study before RYGB surgery. At that time, the national guideline criteria for gastric bypass were a BMI ≥ 40 kg/m^2^, BMI ≥ 35 kg/m^2^ with obesity‐related comorbidity, or a BMI ≥ 30 kg/m^2^ with T2D not manageable with conservative treatment. This cohort has previously been described by Härma et al. (2021), Zhao et al. (2024) and Kaisanlahti et al. (2025) The RYGB surgery and medical follow‐up for patients with obesity with T2D (*n* = 16) and without T2D (*n* = 14) were conducted at Oulu University Hospital, Oulu, Finland (Table S1). The fecal samples collected at baseline and 6 months after the surgery in 2010–2019 and were stored at −80°C. The fecal samples from healthy volunteers (*n* = 9) were collected at one time point. The Ethics Committee of the Northern Ostrobothnia Hospital District, Oulu, Finland reviewed and approved the study protocol (decision number EETTMK:96/2008, EETTMK:103/2018). Each study participant provided written informed consent. Samples collected under clinical trial registered with ClinicalTrials.gov.

### 4.1 Isolation of Extracellular Vesicles

The gut microbiota–derived EVs were isolated according to a previously described the detailed purification protocol (Byts et al. 2023; Kaisanlahti et al. 2023). In brief, frozen fecal samples were melted on ice, 4 g of feces was suspended in 50 mL 1× Dulbecco's PBS (dPBS) for EV protein isolation, 1 g of feces was suspended in 15 mL dPBS for EV RNA isolation. We included two negative controls for EV isolation of pure dPBS. The fecal suspension was centrifuged twice at 14,000 g for 30 min to remove cellular debris and undigested material. The supernatant was filtered twice, first through a 40 μm nylon filter (Corning, USA) then trough a 0.45 μm pore, PES Membrane Vacuum Bottle Filter (1000 mL or 150 mL Thermo Fisher Scientific, USA). Filtrate was concentrated according to manufacturer instructions using Centricon Plus‐70 centrifugal filtering device (Merck Millipore, USA) for EV protein isolation and the Amicon Ultra 15 centrifugal filter unit (Merck Millipore) for EV RNA isolation. The EVs were isolated by size‐exclusion chromatography according to the manufacturer's protocol with the Exo‐Spin Mini Columns (Cell Guidance Systems, UK). Bacterial EVs were enriched from the total mixed population of human and bacterial EVs by iodixanol density gradient ultracentrifugation (OptiPrep, Thermo Fisher Scientific) (Tulkens et al. 2020) by centrifuging at 100,000 g for 15 h. Fractions 6 and 7 were used in further analysis after the iodixaol was removed by washing once with dPBS by centrifuging at 100,000 g for 2,5 h.

### 4.2 Characterization of Extracellular Vesicles

The EV samples were placed on Formvar carbon coated copper glow‐discharged grids and fixed with 1% glutaraldehyde. The negative staining of EVs was done using 0.4% uranyl acetate—2% methylcellulose solutions. TEM imaging (Tecnai G2 Spirit, FEI, USA) was used to confirm the quality of isolation. Negative staining and TEM imaging were performed at the Tissue Imaging Core facility, Biocenter Oulu, University of Oulu, Finland.

Particle sizes and the concentration of samples were estimated in 1:25 diluted samples with NanoSight (NS300, Malvern Pananalytical, USA) using Nanoparticle Tracking Analysis‐software (3.4.4.) Vesicle sizes and concentration were compared between groups by Student's *t*‐test and paired *t*‐test when appropriate.

### 4.3 Protein Isolation and Mass Spectrometry

The EV protein isolation was performed using methanol chloroform precipitation following Friedman (2007), in which all reagent volumes are adjusted by the initial volume of vesicles. Briefly, the vesicles were mixed with sterile water, methanol, and chloroform at a ratio of 1:3:4:1, respectively. The resulting precipitate was washed again adding fourfold volume of methanol and the precipitate was centrifuged to the bottom of the tube. The precipitate was dried and dissolved in 1× Laemly loading buffer (Bio‐Rad, USA) by boiling for 5 min. The samples were loaded to 12% Mini‐Protean TGX (Bio‐Rad) gel, and SDS‐PAGE electrophoresis was performed at 110 V until the samples were completely in the gel (approximately 10 min). The gel was fixed in 50% ethanol and 10% acetic acid solution for 30 min, rinsed with water and then stained overnight with SYPRO Ruby protein gel stain (Thermo Fisher Scientific). Excess dye was removed by washing with 5% acetic acid for 5 min and in double‐distilled water for 15 min. The gel was dried by ethanol for transport. Before mass spectrometry, the samples were in‐gel digested via trypsin, and peptides were dissolved in 0.1% formic acid. The LC‐ESI‐MS/MS analyses were performed on a nanoflow HPLC system (Easy‐nLC1200, Thermo Fisher Scientific) coupled to a Q Exactive HF mass spectrometer (Thermo Fisher Scientific) equipped with a nano‐electrospray ionization source. The mass spectrometry analysis was performed at the Turku Proteomics Facility, University of Turku and Åbo Akademi University, Turku, Finland.

### 4.4 Proteomic Data Analysis

Sequences of proteomic samples were analyzed by PEAKS Studio software (v. 10.6) (Bioinformatics Solutions Inc., USA). For protein and peptide identification, the database search was performed against the Uniprot/Swiss‐Prot and the Uniprot/TrEMBL databases (v. 2022_05) with a parent mass error tolerance of 10.0 ppm, and a fragment mass error tolerance of 0.02 Da; the FDR for both was 1.0%. Top protein filtration incorporated of PEAKS software was used. Proteins were identified to be present if at least one unique peptide with a total coverage of supporting peptides constituted more than 1%. The results are available in the PRIDE protein database (Perez‐Riverol et al. 2022). Proteins presenting in the negative control samples were excluded from the identification results. The fecal EV isolation was designed to enrich microbial EVs (Tulkens et al. 2020; Byts et al. 2023). Yet, the isolated EVs containing human proteins were presented as well for their raw numbers.

### 4.5 Total RNA Isolation From Extracellular Vesicles

We chose to extract total RNA from fecal EVs because limited data are available regarding the presence of genomic DNA in EVs (Zhou et al. 1998; Toyofuku et al. 2018). Total RNA isolation from fecal EVs was performed using the exoRNeasy Serum Plasma Midi Kit (#77044, Qiagen, Germany) according to the manufacturer's protocol with some modifications. We included negative isolation controls to exclude possible contaminants from RNA isolation. Briefly, isolated vesicle samples were diluted to a 1:10 ratio and mixed at a 1:5 ratio of Qiazol and incubated for 5 min to promote the dissolution of nucleoprotein complexes. Roughly a 1:8 ratio of chloroform was added and mixed properly. The samples were centrifuged at 12,000 g for 15 min, and the upper phase was transferred with 2 volumes of pure ethanol to another tube. The sample was added to the RNeasy MinElute spin column and spun for 15 s at 12,000 g, repeated if necessary. RWT buffer was centrifuged through the column within 15 s at 12,000 g and repeated with RPE buffer two times. The membrane was dried by centrifuging for 5 min at full speed. RNA was eluted out to 14 μL of RNase‐free water by centrifuging for 1 min at full speed and repeating by circulating the eluate.

### 4.6 Total DNA Isolation From Feces

Total DNA isolation from feces was performed using the QIAamp Fast DNA Stool Mini Kit (#51604, Qiagen) according to the manufacturer's protocol. Altogether, 200 mg of fecal material was homogenized with 1 mL of InhibitEX buffer and centrifuged at 14,000 g for 1 min to pellet fecal particles. Corresponding weight of dPBS was added as negative controls of DNA isolation. The supernatant and proteinase K were mixed at a ratio of 24:1, respectively. Buffer AL was added to the supernatant at a 1:1 ratio and the samples were incubated for 10 min at 70°C. Altogether, 600 μL of ethanol was added to the lysate, which was then transferred to QIAmp spin column and centrifuged at 14,000 g for 1 min. The columns were washed with buffer AW1 and buffer AW2. The DNA was eluted in 200 μL buffer ATE by centrifuging for 1 min at 14,000 g.

### 4.7 Total RNA Conversion to cDNA, PCR, and 16S rRNA Gene Sequencing

Fecal EV RNA was converted to cDNA for PCR, using the iScript cDNA synthesis kit (#1708890, Bio‐Rad) according to the manufacturer's protocol. The reaction mixture included approximately 200 ng of RNA template, 0.5 μM of 16S rRNA specific primer S‐*‐Univ‐0515‐a‐S‐19 (5′‐GTGCCAGCMGCCGCGGTAA‐3′), and 1× iScript reaction buffer.

The 16S rRNA gene sequencing were performed using total genomic DNA from fecal samples and converted cDNA from EVs as template. The sequencing of the V4–V5 hypervariable region of the 16S rRNA gene from EVs performed using S‐*‐Univ‐0519‐a‐S‐18 (5′CAGCMGCCCGCGGTAATWC‐3′) and S‐D‐Bact‐0907‐a‐A‐20 (5′‐CCGTCAATTCCTTTRAGTTT‐3′) primers. A forward primer was included with the Ion Torrent adaptor sequence, and individual barcodes were assigned to for each sample to enable pooling during sequencing. The reverse primer initially contained the Ion Torrent adapter sequence trP1. PCR was performed according to manufacturer's protocol in duplicate 20 μL reactions with Flash High Fidelity PCR master mix (Thermo Fisher Scientific), 1.0 μM of forward and reverse primers, and 10 ng of genomic DNA or 4 μL of converted cDNA as a template. The sequencing run included two negative controls of sterile water as well as three mock community controls using Zymobiomics microbial mock community standard II (Zymo Research, USA) diluted at 1:100. An Applied Biosystems Veriti 96‐Well Thermal Cycler (Thermo Fisher Scientific) was used for PCR reactions. The program included an initialization phase of 3 min at 98°C. After the initial 3 min denaturation at 98°C, the following conditions were applied to amplify the target region: 98°C for 10 s, 64°C for 10 s, and 72°C for 30 s. Genomic DNA samples were amplified for 24 cycles, cDNA for 28 cycles, and mock community samples for 28 cycles. The final extension step was carried out at 72°C for 5 min. Duplicate reactions were combined after amplification and purified with the Agencourt AMPure XP PCR purification system (Beckman Coulter Inc., USA). Samples were pooled together based on the assigned appropriate equimolar ratio using Bioanalyzer with DNA‐1000 kit (Agilent, USA). The pooled libraries were purified again with the AMPure XP, analyzed with Bioanalyzer, and the libraries' DNA concentration were measured with a Quant‐iT PicoGreen assay kit (Invitrogen, Thermo Fisher Scientific). DNA sequencing was performed at Biocenter Oulu Sequencing Center (University of Oulu, Finland) with the Ion Torrent PGM sequencer (Thermo Fisher Scientific). The sequencing run was performed with an Ion PGM Hi‐Q View OT2 template kit using a 400 bp templating program, an Ion PGM Hi‐Q View sequencing kit with 850 cycles, and a 316 v2 chip.

### 4.8 16 s rRNA Gene Sequence Analysis

The 16 s rRNA gene sequence analysis was performed in R (v. 4.4.2) and RStudio (v. 2024.04.0 + 735). High‐quality sequencing reads (maxEE = 2, truncQ = 2) were chosen, truncated after 240 bp, and chimeric reads were filtered out using DADA2 (v.1.34.0) (Callahan et al. 2016). Counts through to filtering process are available in Tables S11 and S12. Negative controls from EV isolation, RNA/DNA isolation and sequencing containing contaminant reads were filtered out using the decontam R package (version 1.26.0) (Davis et al. 2018). A prevalence‐based method was employed in which the frequency of the taxon in the control samples is compared to its the frequency of the taxon in true samples, and the taxon is deemed a contamination if the prevalence in control samples exceeds a threshold. The threshold was set to 0.1 and contaminant reads were filtered out of the dataset. Analyses were performed in R using the Mia and MiaViz packages (v. 1.17.4) (Ernst et al. 2023). Relative abundances in the phylum and genus levels were estimated using the observed ASVs, and the taxonomy was assigned against SILVA (v. 138.1) (Quast et al. 2013). The data for statistical analysis were rarefied to a minimum sample read count with iteration. Differentially abundant taxa were estimated using Analysis of Composition of Microbiomes with Bias Correction (ANCOM‐BC) using package ANCOMBC (v.2.8.1) (Lin and Peddada 2020). For the method of correcting *p*‐values was used FDR (Benjamini‐Hochberg). Lcf, log fold change (natural log), *W* = lfc/se (se = standard error of lfc), and *p*‐values were obtained from two‐sided Z‐test using *W* and *q*‐values denote FDR adjusted *p*‐values. ANCOM‐BC was performed in paired when appropriate. The Shannon index was used to estimate alpha diversity metrics, and differences in alpha diversity between groups were estimated with Wilcoxon rank sum test with FDR‐correction. In paired alpha diversity analysis, Wilcoxon signed rank with FDR was used to estimate statistical significance. Community similarity (beta diversities) was visualized as a Bray‐Curtis distance using Principal Coordinate Analysis (PCoA). The significance of differences in community composition was estimated by Permutational Multivariate Analysis Of Variance (PERMANOVA) with multidimensional FDR‐correction using the vegan R‐package (v.2.7–1) (Oksanen et al. 2022). PERMANOVA performed also paired when appropriate.

### 4.9 Sample Group Classification Using Machine Learning

As another means of exploring differences between the sample groups, the groups for each sample were classified by machine learning, performed with QIIME2 (v. 2023.5) (Bolyen et al. 2019). The demultiplexed reads were preprocessed as described above. Contaminant reads were filtered out of the data utilizing data from previously mentioned experimental negative controls and decontam R package as described above. The resulting feature table was used for sample group classification. To build a model for classification, the Random Forest (RF) nested cross‐validation (NCV) method was used, in which the dataset was divided into training (80%) and test datasets (20%), and subsequent training and testing of the classifier. The process was repeated until each sample appeared once in the test dataset. To minimize overfitting, model performance was evaluated using K‐fold cross‐validation. The number of trees used in the prediction was set to 100. The number of k‐fold cross‐validations was set to 5. Sample group classifications were made on the gastric bypass samples, and samples were classified into either the baseline–group (0 months, *n* = 30) or the groups after surgery (6 months, *n* = 30).

## Author Contributions

**Jenni Hekkala:** investigation, methodology, formal analysis, data curation, visualization, writing – original draft. **Jenni Turunen:** methodology, formal analysis, visualization, writing – review and editing. **Pande Putu Erawijantari:** software, writing – review and editing. **Leo Lahti:** software, methodology, writing – review and editing. **Anatoliy Samoylenko:** methodology. **Anna Kaisanlahti:** formal analysis, methodology, visualization, writing – review and editing. **Seppo Vainio:** methodology. **Nikke Virtanen:** investigation. **Mysore V. Tejesvi:** methodology, visualization, writing – review and editing. **Sonja Karikka:** visualization, formal analysis. **Terhi Ruuska‐Loewald:** conceptualization, methodology, supervision, funding acquisition, writing – review and editing. **Genevieve Bart:** methodology, writing – review and editing. **Justus Reunanen:** conceptualization, methodology, supervision, funding acquisition, writing – review and editing. **Vesa Koivukangas:** conceptualization, funding acquisition, writing – review and editing. **Janne Hukkanen:** conceptualization, funding acquisition, writing – review and editing.

## Disclosure

Authors declare no Artificial Intelligence generated content or large language models used in this manuscript.

## Ethics Statement

The Ethics Committee of the Northern Ostrobothnia Hospital District, Oulu, Finland reviewed and approved the study protocol (decision number EETTMK:96/2008, EETTMK:103/2018).

## Consent

Each study participant provided written informed consent.

## Conflicts of Interest

The authors declare no conflicts of interest.

## Supporting information


**Data S1:** Supporting information.

## Data Availability

The 16S rRNA data that support the findings of this study are openly available in Genbank at https://www.ncbi.nlm.nih.gov/genbank/, reference number PRJNA1041054. Proteomic data that support the findings of this study are openly available in PRIDE at https://www.ebi.ac.uk/pride/, reference number PXD050033. Scripts used in data analysis are available in Zenodo https://zenodo.org/, with doi: https://10.5281/zenodo.15662584.
