# Supplementary material for: Gut Microbiota‐Derived Extracellular Vesicles in Patients With Obesity Undergoing Gastric Bypass Surgery
Source: Mol Microbiol. 2026 Mar 12;125(5):412–25. doi: 10.1111/mmi.70064 (PMC13135909; doi:10.1111/mmi.70064)
Supplement: Supplementary file 1 — Data S1: Supporting information. [file MMI-125-412-s001.docx]

# Gut microbiota -derived extracellular vesicles in patients with obesity undergoing gastric bypass surgery

Jenni Hekkala*^1,2^, Anna Kaisanlahti^1,2^, Mysore V. Tejesvi^2,3^, Jenni Turunen^2,3^, Nikke Virtanen^1,2^, Sonja Karikka^1,2,4^, Pande Putu Erawijantari^5^, Anatoliy Samoylenko^4^, Genevieve Bart^4^, Seppo Vainio^4,6^, Leo Lahti^5^, Janne Hukkanen^7^, Terhi Ruuska-Loewald^2,3,8^, Vesa Koivukangas^3^ and Justus Reunanen ^1,2^

Affiliations:

1. Research Unit of Translational Medicine, University of Oulu, Oulu, Finland

2. Biocenter Oulu, University of Oulu, Oulu, Finland

3. Research Unit of Clinical Medicine, University of Oulu, Oulu, Finland

4. Laboratory of Developmental Biology, Disease Networks Research Unit, Faculty of Biochemistry and Molecular Medicine, University of Oulu, Oulu, Finland

5. Department of Computing, University of Turku, Turku, Finland.

6. Kvantum Institute, University of Oulu, Oulu, Finland

7. Research Unit of Biomedicine and Internal Medicine, University of Oulu, and Medical Research Center Oulu, Oulu University Hospital and University of Oulu, Oulu, Finland

8. Department of Pediatrics and Adolescent Medicine, Oulu University Hospital, Oulu, Finland

*Corresponding author.

Name: Jenni Hekkala

Email: [jenni.hekkala@oulu.fi](mailto:jenni.hekkala@oulu.fi)

ORCID: 0000-0002-5826-1196

Adress: University of Oulu

Aapistie 5, P.O. box 5281

90014 University of Oulu, Finland

Tel: +358 40 4157388

## **Supplementary information**


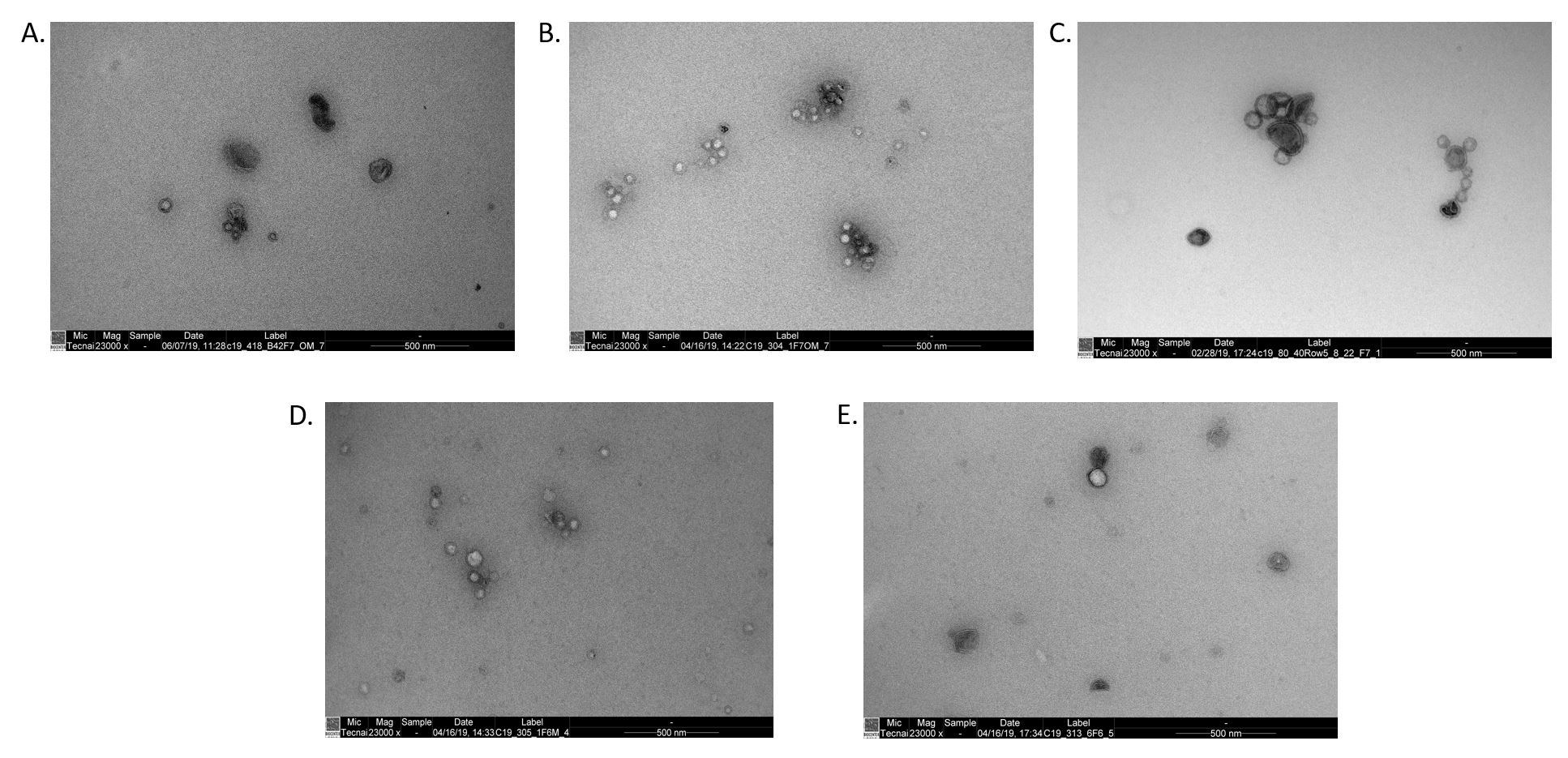


**Figure S1** Representative transmission election microscopy (TEM) images of vesicles in **A.** volunteer; **B.** patients with obesity without T2D at baseline; **C.** patients with obesity and T2D at baseline; **D.** patients with obesity without T2D after surgery; **E.** patients with obesity and T2D after surgery

**TABLE S1** Clinical characteristic of study aprticipants, BMI= body mass index, ΔBW=change of bodyweight in kilograms from baseline to 6 months after surgery. NA= not applicable

|  | All patients with obesity  (ALL_OB)  n=30 | w/o T2D  (OB)  n=14 | w T2D  (OB_T2D)  n=16 | Healthy volunteers  n=9 |
| --- | --- | --- | --- | --- |
| Women  n (%) | 21 (70) | 11 (79) | 10 (62,5) | 7 (80) |
| Age  Mean (SD) | 47.3 (8.8) | 44.5 (9.0) | 49.8 (8.1) | 46.3 (11.1) |
| BMI  Mean (SD) | 44.5 (5.7) | 47.5 (5.6) | 41.8 (4.3) | 26.1 (4.4) |
| ΔBW (kg)  Mean (SD) | -27.2 (7.8) | -27.8 (7.0) | -26.6 (8.6) | NA |

**
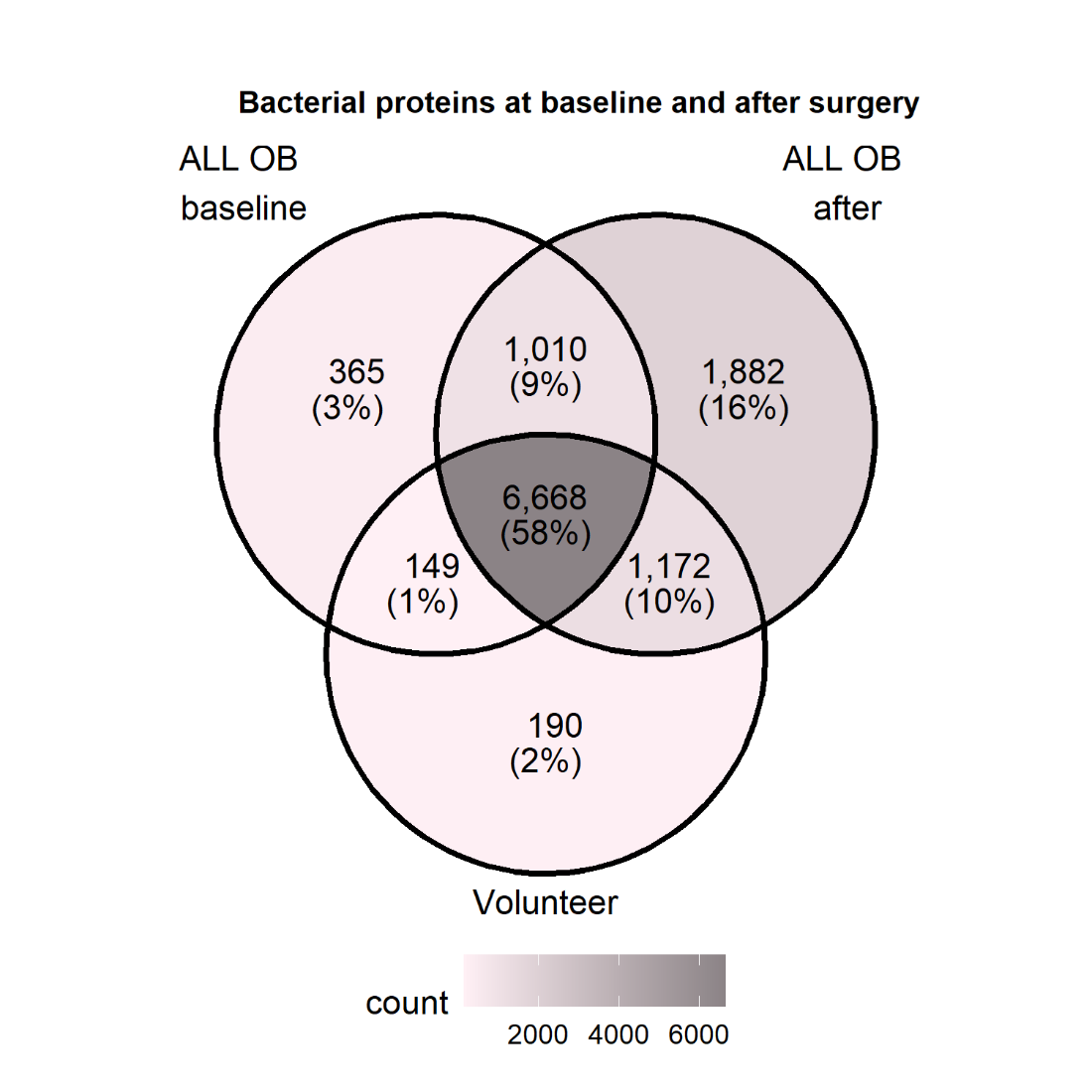
**

**Figure S2** Venn diagram, Healthy volunteers and all patients with obesity at baseline and after gasric bypass surgery. ALL OB baseline= all patients with obesity at baseline; ALL OB after= all patients with obesity 6 months after surgery


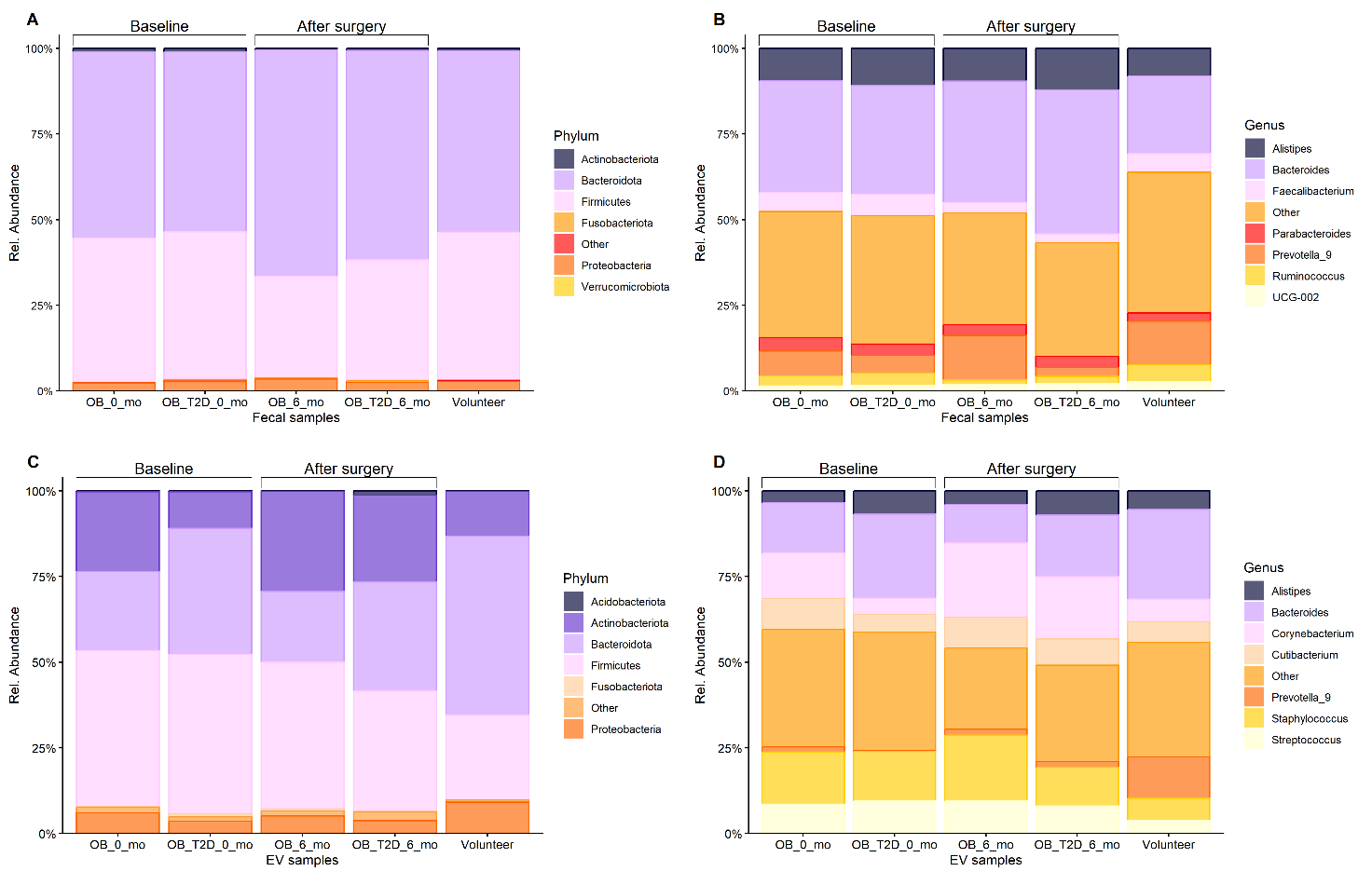


**Figure S3** Relative abundance of groups of groups from **A.** gut microbiota 16S rDNA at the phylum level; **B.** at the genus level; **C.** gut microbiota-derived EVs 16s rRNA in phylum level; **D.** in genus level. OB_0_mo= patients with obesity without T2D at baseline; OB_T2D_0_mo= patients with obesity and T2D at baseline; OB_6_mo= patients with obesity without T2D 6 months after surgery; OB_T2D_6_mo= patients with obesity and T2D 6 months after surgery.

**TABLE S2** The 6 most abundant phyla in gut microbiota and gut microbiota-derived EVs. OB_0_mo= patients with obesity without T2D at baseline; OB_T2D_0_mo= patients with obesity and T2D at baseline; OB_6_mo= patients with obesity without T2D 6 months after surgery; OB_T2D_6_mo= patients with obesity and T2D 6 months after surgery.

| Phyla | FE_OB_0mo | FE_OB_T2D_0mo | FE_OB_6mo | FE_OB_T2D_6mo | FE_volunteer |
| --- | --- | --- | --- | --- | --- |
| Bacteroidota | 0.543 | 0.526 | 0.66 | 0.61 | 0.53 |
| Firmicutes | 0.421 | 0.431 | 0.297 | 0.353 | 0.432 |
| Proteobacteria | 0.023 | 0.027 | 0.034 | 0.024 | 0.027 |
| Actinobacteriota | 0.010 | 0.010 | 0.0044 | 0.0062 | 0.0067 |
| Fusobacteriota | 0.0007 | 0.0053 | 0.003 | 0.0049 | 0.0003 |
| Verrucomicrobiota | 0.00008 | 0.00013 | 0.0003 | 0.0002 | 0.0006 |
| Other | 0.00098 | 0.0011 | 0.0014 | 0.00052 | 0.0034 |
| Phyla | **EV_OB_0mo** | **EV_OB_T2D_0mo** | **EV_OB_6mo** | **EV_OB_T2D_6mo** | **EV_volunteer** |
| Bacteroidota | 0.231 | 0.368 | 0.206 | 0.319 | 0.524 |
| Firmicutes | 0.455 | 0.466 | 0.429 | 0.351 | 0.246 |
| Actinobacteriota | 0.233 | 0.107 | 0.293 | 0.253 | 0.131 |
| Proteobacteria | 0.061 | 0.036 | 0.051 | 0.037 | 0.091 |
| Deinococcota | 0.0037 | 0.0022 | 0.0012 | 0.0023 | 0.0024 |
| Fusobacteriota | 0.0031 | 0.0087 | 0.005 | 0.0021 | 0.0011 |
| Other | 0.014 | 0.014 | 0.014 | 0.037 | 0.0044 |
|  | |  |  |  |  |

**TABLE S3** The 6 most abundant genera in fecal samples and EVs. OB_0_mo= patients with obesity without T2D at baseline; OB_T2D_0_mo= patients with obesity and T2D at baseline; OB_6_mo= patients with obesity without T2D 6 months after surgery; OB_T2D_6_mo= patients with obesity and T2D 6 months after surgery.

| **Genus** | **FE_OB_0mo** | **FE_OB_T2D_0mo** | **FE_OB_6mo** | **FE_OB_T2D_6mo** | **FE_volunteer** |
| --- | --- | --- | --- | --- | --- |
| Bacteroides | 0.308 | 0.298 | 0.33 | 0.401 | 0.208 |
| Alistipes | 0.09 | 0.102 | 0.09 | 0.115 | 0.074 |
| Prevotella_9 | 0.068 | 0.049 | 0.12 | 0.025 | 0.117 |
| Faecalibacterium | 0.053 | 0.059 | 0.03 | 0.025 | 0.050 |
| Parabacteroides | 0.038 | 0.030 | 0.029 | 0.031 | 0.022 |
| Ruminococcus | 0.0281 | 0.034 | 0.011 | 0.019 | 0.044 |
| Other | 0.401 | 0.411 | 0.372 | 0.362 | 0.458 |
| **Genus** | **EV_OB_0mo** | **EV_OB_T2D_0mo** | **EV_OB_6mo** | **EV_OB_T2D_6mo** | **EV_volunteer** |
| Bacteroides | 0.144 | 0.232 | 0.105 | 0.167 | 0.259 |
| Corynebacterium | 0.13 | 0.044 | 0.201 | 0.168 | 0.065 |
| Staphylococcus | 0.149 | 0.137 | 0.179 | 0.104 | 0.063 |
| Streptococcus | 0.084 | 0.090 | 0.09 | 0.075 | 0.038 |
| Cutibacterium | 0.088 | 0.049 | 0.084 | 0.071 | 0.059 |
| Alistipes | 0.033 | 0.065 | 0.036 | 0.065 | 0.053 |
| Other | 0.334 | 0.363 | 0.275 | 0.313 | 0.442 |

**TABLE S4** ANCOM-BC analysis, statistically significant phyla of gut microbiota from all patients with obesity compared at baseline and after surgery, lfc=log-fold-change of relative abundance, q-value= FDR adjusted p-value.

| **taxon** | **lfc_ALL_OB_after** | **q_ALL_OB_after** |
| --- | --- | --- |
| Actinobacteriota | -0.7476 | 0.046 |
| Verrucomicrobiota | 0.9484 | 0.046 |

**TABLE S5** ANCOM-BC analysis, statistically significant genera of gut microbiota from all patients with obesity compared at baseline and after surgery, lfc=log-fold-change of relative abundance, q-value= FDR adjusted p-value.

| **taxon** | **lfc_ALL_OB_after** | **q_ALL_OB_after** |
| --- | --- | --- |
| Streptococcus | 1.0739 | 0.0035 |
| Veillonella | 1.3206 | 0.0035 |
| Lachnoclostridium | 1.0716 | 0.013 |
| Listeria | -0.7738 | 0.018 |
| Staphylococcus | -0.7654 | 0.018 |
| NK4A214 group | 1.2326 | 0.018 |
| [Eubacterium] siraeum group | -0.7654 | 0.019 |
| Erysipelotrichaceae UCG-003 | -0.6396 | 0.022 |
| Rhodococcus | -0.6645 | 0.022 |
| Cutibacterium | -0.7810 | 0.022 |
| [Eubacterium] eligens group | 0.9185 | 0.022 |
| Corynebacterium | -0.7729 | 0.027 |
| Lactobacillus | -0.6585 | 0.027 |
| Lachnospiraceae UCG-004 | 1.0149 | 0.027 |
| Prevotellaceae NK3B31 group | -0.7763 | 0.036 |
| Anaerococcus | -0.6524 | 0.037 |
| Escherichia-Shigella | 0.7939 | 0.038 |
| Megasphaera | 1.7627 | 0.041 |
| UCG-003 | 0.9349 | 0.043 |

**TABLE S6** ANCOM-BC analysis, statistically significant phyla of gut microbiota from patients with obesity and without T2D compared at baseline and after surgery, lfc=log-fold-change of relative abundance, q-value= FDR adjusted p-value.

| **taxon** | **lfc_ ALL_OB_baseline** | **q_ ALL_OB_baseline** |
| --- | --- | --- |
| Actinobacteriota | -1.9276 | 0.0068 |

**TABLE S7** ANCOM-BC analysis, statistically significant genus of gut microbiota from volunteers compared to all patients with obesity at baseline, lfc=log-fold-change of relative abundance, q-value= FDR adjusted p-value.

| **taxon** | **lfc_ ALL_OB_baseline** | **q_ ALL_OB_baseline** |
| --- | --- | --- |
| Allorhizobium-Neorhizobium-Pararhizobium-Rhizobium | -0.7507 | 0.027 |

**TABLE S8** ANCOM-BC analysis, statistically significant genus of gut microbiota from volunteers compared to all patients with obesity after surgery, lfc=log-fold-change of relative abundance, q-value= FDR adjusted p-value.

| **taxon** | **lfc_ ALL_OB_after** | **q_ ALL_OB_after** |
| --- | --- | --- |
| Veillonella | 1.8855 | 0.00054 |
| Lachnoclostridium | 1.6920 | 0.0012 |
| Streptococcus | 1.3570 | 0.0014 |

**TABLE S9** ANCOM-BC analysis, statistically significant genera of gut microbiota-derived EVs from volunteers compared to all patients with obesity at baseline, lfc=log-fold-change of relative abundance, q-value= FDR adjusted p-value.

| **taxon** | **lfc_ALL_OB_baseline** | **q_ ALL_OB_baseline** |
| --- | --- | --- |
| Prevotella_9 | -3.3418 | 0.011 |

**TABLE S10** ANCOM-BC analysis, statistically significant genera of gut microbiota-derived EVs from volunteers compared to all patients with obesity after surgery, lfc=log-fold-change of relative abundance, q-value= FDR adjusted p-value.

| **taxon** | **lfc_ALL_OB_after** | **q_ ALL_OB_after** |
| --- | --- | --- |
| Prevotella_9 | -2.7328 | 0.040 |
| Lactococcus | -1.1634 | 0.043 |

**
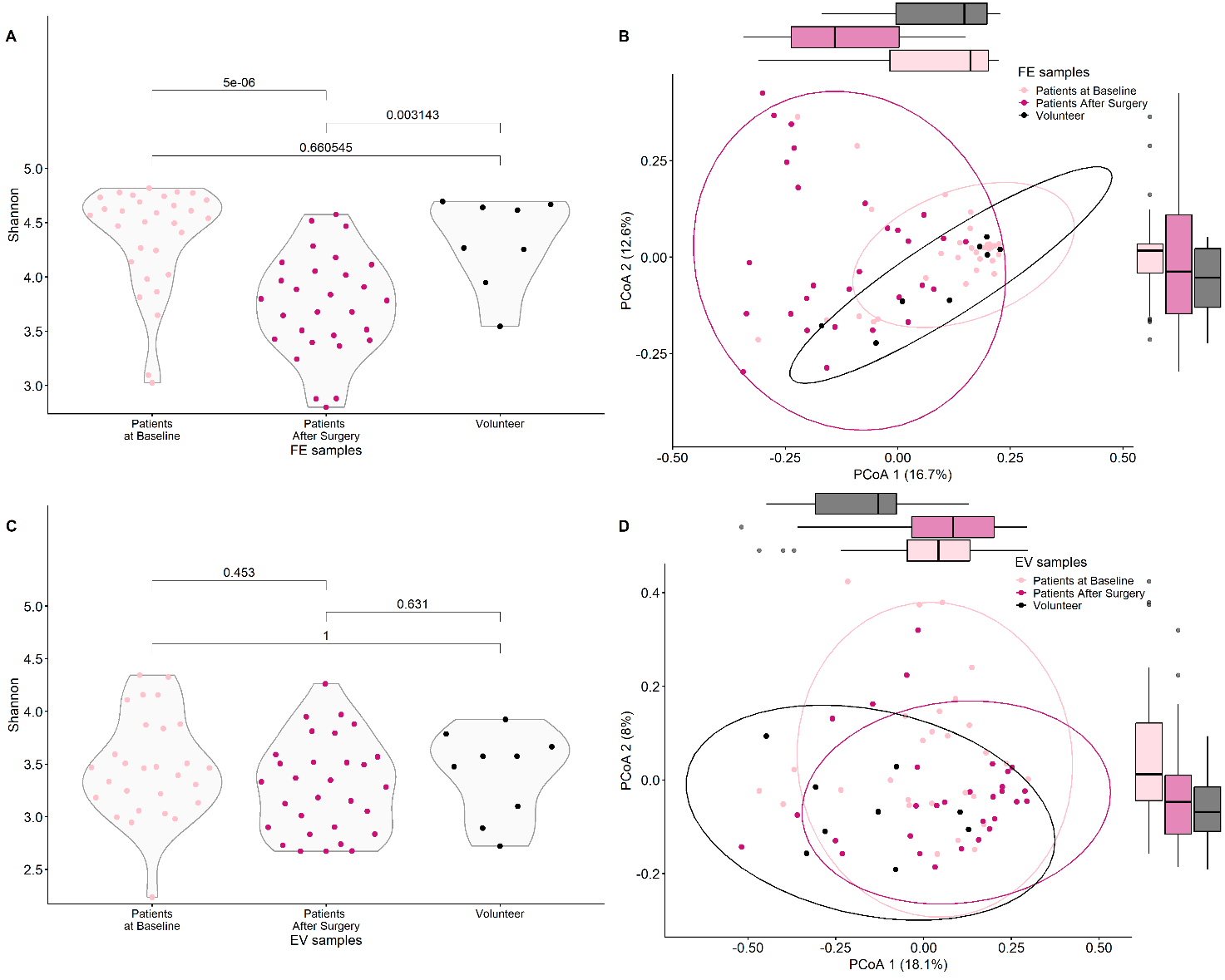
**

**Figure S4. A.** Alpha diversity violin plot of gut microbiota by Shannon index, statistical significance by Wilcoxon rank sum with FDR correction **B.** PCoA- plot of gut microbiota by Bray-Curtis dissimilarity matrix, statistical significance by PERMANOVA(Effect size R^2^= 0.09255, *p*= 0.001) **C.** Alpha diversity violin plot of gut microbiota–derived EVs by Shannon index, statistical significance by Wilcoxon rank sum with FDR correction **D.** PCoA- plot of gut microbiota–derived EVs by Bray-Curtis dissimilarity matrix, statistical significance by PERMANOVA (Effect size R^2^= 0.06002, *p*= 0.002). ALL_OB_baseline= all patients with obesity at baseline (FE: *n*=30, EV: *n*=29); ALL_OB_after= all patients with obesity 6 months after surgery (FE: *n*=29, EV: *n*=30). Volunteers (FE: *n*=8, EV: *n*=9).

**TABLE S11** Read counts of EV samples trough DADA2. Low quality samples, controls and duplicates removed after decontam.

| **track_EV_samples** | **input (reads)** | **filtered**  **(reads)** | **denoised**  **(reads)** | **ASV** |
| --- | --- | --- | --- | --- |
| 0M01_TATATCACT_L001_R1_001.fastq.gz | 23698 | 12723 | 12493 | 12493 |
| 0M02_TGTGTCGTC_L001_R1_001.fastq.gz | 22085 | 11334 | 11205 | 11157 |
| 0M03_ACTCTCTCT_L001_R1_001.fastq.gz | 91513 | 43678 | 43161 | 42274 |
| 0M04_AGTGTGCTC_L001_R1_001.fastq.gz | 20098 | 10268 | 10243 | 10175 |
| 0M05_CGAGAAGTC_L001_R1_001.fastq.gz | 20573 | 11931 | 11765 | 11731 |
| 0M06_TTATACATC_L001_R1_001.fastq.gz | 22207 | 12673 | 12586 | 12495 |
| 0M09_GCACAGCTC_L001_R1_001.fastq.gz | 24556 | 13809 | 13684 | 13628 |
| 0M10_ATAGCACGG_L001_R1_001.fastq.gz | 27378 | 15112 | 15048 | 15015 |
| 0M12_CAATCTCTC_L001_R1_001.fastq.gz | 26571 | 15290 | 15183 | 15173 |
| 0M13_CGAAGAGCT_L001_R1_001.fastq.gz | 28014 | 17163 | 17035 | 17035 |
| 0M15_TTACGCACT_L001_R1_001.fastq.gz | 23939 | 12588 | 12554 | 12494 |
| 0M16_CCATGTATC_L001_R1_001.fastq.gz | 23991 | 10961 | 10853 | 10844 |
| 0M17_ACAGTATGG_L001_R1_001.fastq.gz | 29543 | 18492 | 18294 | 18278 |
| 0M18_ACAGTTATC_L001_R1_001.fastq.gz | 23592 | 12688 | 12605 | 12600 |
| 0M19_CGCTACAGG_L001_R1_001.fastq.gz | 34517 | 18258 | 18097 | 18097 |
| 0M20_TGCTAGTTC_L001_R1_001.fastq.gz | 23829 | 13775 | 13692 | 13642 |
| 0M21_AACACATCT_L001_R1_001.fastq.gz | 36352 | 20284 | 20111 | 20041 |
| 0M22_ATCTCCTCT_L001_R1_001.fastq.gz | 14678 | 7445 | 7428 | 7272 |
| 0M23.0_TACTGGCTC_L001_R1_001.fastq.gz | 23913 | 13592 | 13514 | 13514 |
| 0M23_^CGCTACAGG_L001_R1_001.fastq.gz | 71207 | 59014 | 58863 | 58689 |
| 0M24_AACTGTATC_L001_R1_001.fastq.gz | 26244 | 14839 | 14807 | 14751 |
| 0M25_TACTGTAGG_L001_R1_001.fastq.gz | 4220 | 2396 | 2377 | 2377 |
| 0M26_GACTGTACT_L001_R1_001.fastq.gz | 26914 | 14627 | 14557 | 14492 |
| 0M27_GACGTATCT_L001_R1_001.fastq.gz | 22905 | 13196 | 13128 | 13128 |
| 0M28_GACGTGCTC_L001_R1_001.fastq.gz | 23933 | 12625 | 12570 | 12539 |
| 0M29_GTGTGATCT_L001_R1_001.fastq.gz | 25342 | 15360 | 15267 | 15186 |
| 0M30_GCGATCATC_L001_R1_001.fastq.gz | 26816 | 14562 | 14511 | 14511 |
| 0M32_GATGCACTC_L001_R1_001.fastq.gz | 23702 | 10286 | 10200 | 10175 |
| 0M33_GATGCGTCT_L001_R1_001.fastq.gz | 30331 | 16313 | 16230 | 16211 |
| 0M34_TACGTTATC_L001_R1_001.fastq.gz | 23094 | 13855 | 13723 | 13723 |
| 0M35_CCGTACATC_L001_R1_001.fastq.gz | 23313 | 12106 | 11898 | 11893 |
| 0M40_TCGGCACTC_L001_R1_001.fastq.gz | 24865 | 14228 | 14093 | 14078 |
| 0M41_CTGACATTC_L001_R1_001.fastq.gz | 37219 | 20453 | 20192 | 20179 |
| 0M42_^TGCTAGTTC_L001_R1_001.fastq.gz | 40413 | 32438 | 32262 | 32262 |
| 0M43_ACGCGACCT_L001_R1_001.fastq.gz | 25456 | 15731 | 15689 | 15407 |
| 0M44_GCGCGACTC_L001_R1_001.fastq.gz | 27601 | 15688 | 15575 | 15455 |
| 0M45_TCGATACCT_L001_R1_001.fastq.gz | 43660 | 21597 | 21325 | 21204 |
| 0M46_CTGGTATCT_L001_R1_001.fastq.gz | 36791 | 15145 | 14957 | 14942 |
| 0M47_TCGATGTTC_L001_R1_001.fastq.gz | 42700 | 22291 | 22056 | 21167 |
| 6M01_GTCAATCT_L001_R1_001.fastq.gz | 17217 | 9709 | 9666 | 9657 |
| 6M02_CGTCAGCCT_L001_R1_001.fastq.gz | 164314 | 92816 | 92636 | 92219 |
| 6M03_TGTACATGG_L001_R1_001.fastq.gz | 33047 | 20072 | 19868 | 19868 |
| 6M04_CATGCCACT_L001_R1_001.fastq.gz | 20173 | 11492 | 11403 | 11379 |
| 6M05_AGTACGCCT_L001_R1_001.fastq.gz | 24813 | 13953 | 13818 | 13814 |
| 6M06_AATCGCACT_L001_R1_001.fastq.gz | 21695 | 11736 | 11564 | 11501 |
| 6M09_AATCGTGTC_L001_R1_001.fastq.gz | 35002 | 18395 | 18173 | 18169 |
| 6M10_TCTCTAACT_L001_R1_001.fastq.gz | 29852 | 17830 | 17664 | 17644 |
| 6M12_AATATACTC_L001_R1_001.fastq.gz | 26586 | 13775 | 13668 | 13663 |
| 6M13_CGTGTCGCT_L001_R1_001.fastq.gz | 34597 | 17927 | 17771 | 17702 |
| 6M14_GCACACGAA_L001_R1_001.fastq.gz | 30385 | 17204 | 17019 | 17019 |
| 6M15_TCACAGCAA_L001_R1_001.fastq.gz | 37369 | 20975 | 20785 | 20746 |
| 6M16_CTATAGTAA_L001_R1_001.fastq.gz | 24036 | 15493 | 15424 | 15424 |
| 6M17_CACCAGCTC_L001_R1_001.fastq.gz | 37068 | 18607 | 18457 | 18457 |
| 6M18_ACACAGCCT_L001_R1_001.fastq.gz | 21948 | 12830 | 12774 | 12774 |
| 6M19_CTCAATCTC_L001_R1_001.fastq.gz | 23497 | 14145 | 14073 | 14065 |
| 6M20.0_^GGCTATGCT_L001_R1_001.fastq.gz | 61 | 2 | 1 | 1 |
| 6M20_^AACACATCT_L001_R1_001.fastq.gz | 19747 | 16607 | 16504 | 16481 |
| 6M21.0_CTCTCAATC_L001_R1_001.fastq.gz | 21977 | 12309 | 12215 | 12188 |
| 6M21_^ATCTCCTCT_L001_R1_001.fastq.gz | 54630 | 47205 | 46992 | 46923 |
| 6M22_CACACATGG_L001_R1_001.fastq.gz | 24099 | 14142 | 14037 | 14014 |
| 6M23_CACTGATTC_L001_R1_001.fastq.gz | 28625 | 16982 | 16877 | 16841 |
| 6M24_GACTGCGTC_L001_R1_001.fastq.gz | 14121 | 7772 | 7739 | 7698 |
| 6M25_GGCATACTC_L001_R1_001.fastq.gz | 25983 | 14911 | 14858 | 14842 |
| 6M26_GTCTCTCCT_L001_R1_001.fastq.gz | 22161 | 13999 | 13803 | 13803 |
| 6M27_TACGTATGG_L001_R1_001.fastq.gz | 23831 | 14864 | 14752 | 14740 |
| 6M28_GTCCTCTCT_L001_R1_001.fastq.gz | 20621 | 12298 | 12249 | 12161 |
| 6M30_AGCATGTTC_L001_R1_001.fastq.gz | 23787 | 13858 | 13773 | 13756 |
| 6M32_TGCATGTGG_L001_R1_001.fastq.gz | 24166 | 11766 | 11672 | 11666 |
| 6M33_CACGTTACT_L001_R1_001.fastq.gz | 22896 | 12498 | 12415 | 12399 |
| 6M34_TGCATTGCT_L001_R1_001.fastq.gz | 22193 | 12435 | 12386 | 12210 |
| 6M40_ATGCAATCT_L001_R1_001.fastq.gz | 24178 | 12011 | 11954 | 11696 |
| EVCON1_^ACAGTATGG_L001_R1_001.fastq.gz | 11274 | 9739 | 9579 | 9546 |
| EVCON2_^ACAGTTATC_L001_R1_001.fastq.gz | 6552 | 5578 | 5446 | 5432 |
| negpcr1_^AGACCAGTC_L001_R1_001.fastq.gz | 25 | 12 | 1 | 1 |
| negpcr3_^GTGACTACT_L001_R1_001.fastq.gz | 117 | 9 | 2 | 2 |
| negpcr4_^CAGAGGACT_L001_R1_001.fastq.gz | 244 | 46 | 25 | 25 |
| RNACON_^ACCAGAGCT_L001_R1_001.fastq.gz | 22102 | 18701 | 18596 | 18522 |

**TABLE S12** Read counts of fecal samples trough DADA2. Low quality samples, controls and duplicates removed after decontam.

| **track_FE_samples** | **input (reads)** | **filtered**  **(reads)** | **denoised**  **(reads)** | **ASV** |
| --- | --- | --- | --- | --- |
| 087-001_^AGTTGCGTC_L001_R1_001.fastq.gz | 25012 | 20321 | 19782 | 19564 |
| 087-002_^CATCGGTCT_L001_R1_001.fastq.gz | 27363 | 22333 | 21877 | 21786 |
| 087-003_^TCTCTGGTC_L001_R1_001.fastq.gz | 28512 | 22797 | 22237 | 21974 |
| 087-004_^CATATGTGG_L001_R1_001.fastq.gz | 24492 | 19590 | 19122 | 18976 |
| 087-005_^TGTGTTACT_L001_R1_001.fastq.gz | 27355 | 22511 | 22012 | 21593 |
| 087-006_^ACTCTTCTC_L001_R1_001.fastq.gz | 26433 | 21437 | 21074 | 21024 |
| 087-009_^TATATTGTC_L001_R1_001.fastq.gz | 37619 | 31197 | 30768 | 30481 |
| 087-010_^TGAGAAGCT_L001_R1_001.fastq.gz | 28460 | 23063 | 22646 | 22626 |
| 087-012_^GCACATAGG_L001_R1_001.fastq.gz | 27279 | 22838 | 22519 | 22493 |
| 087-013_^CCACATACT_L001_R1_001.fastq.gz | 23325 | 18781 | 18382 | 18323 |
| 087-014_^TTATATGCT_L001_R1_001.fastq.gz | 25691 | 20948 | 20520 | 20366 |
| 087-015_^GTAGCCATC_L001_R1_001.fastq.gz | 25492 | 19159 | 18667 | 18550 |
| 087-016_^ATAGCCACT_L001_R1_001.fastq.gz | 28650 | 21563 | 21122 | 21031 |
| 087-017_^GTAGCGTGG_L001_R1_001.fastq.gz | 27803 | 21532 | 21131 | 21053 |
| 087-018_^ACAACTACT_L001_R1_001.fastq.gz | 31370 | 25945 | 25351 | 25041 |
| 087-019_^GCATGATTC_L001_R1_001.fastq.gz | 33508 | 27460 | 27034 | 26965 |
| 087-020_^TTACGTGTC_L001_R1_001.fastq.gz | 25019 | 20564 | 19983 | 19700 |
| 087-021_^ATAATCATC_L001_R1_001.fastq.gz | 27347 | 22032 | 21579 | 21401 |
| 087-022_^GTAATCACT_L001_R1_001.fastq.gz | 31353 | 24433 | 24054 | 23950 |
| 087-023_^TAACTCTCT_L001_R1_001.fastq.gz | 20534 | 15786 | 15406 | 15399 |
| 087-024_^GCAGTTACT_L001_R1_001.fastq.gz | 29394 | 23137 | 22675 | 22462 |
| 087-025_^ACGTACAGG_L001_R1_001.fastq.gz | 29775 | 24997 | 24461 | 24314 |
| 087-026_^CAGAGAGTC_L001_R1_001.fastq.gz | 35216 | 28026 | 27703 | 27125 |
| 087-027_^ATGTGATTC_L001_R1_001.fastq.gz | 29270 | 23899 | 23338 | 23144 |
| 087-028_^GTGTGGCTC_L001_R1_001.fastq.gz | 31218 | 25402 | 24908 | 24720 |
| 087-029_^CTGTGTACT_L001_R1_001.fastq.gz | 41871 | 33861 | 33483 | 33041 |
| 087-030_^GAGTTAGCT_L001_R1_001.fastq.gz | 33113 | 28415 | 28023 | 27921 |
| 087-032_^TGAGAGATC_L001_R1_001.fastq.gz | 41063 | 32776 | 32275 | 32115 |
| 087-033_^AGACCGACT_L001_R1_001.fastq.gz | 28882 | 23892 | 23578 | 23465 |
| 087-034_^GCAACTATC_L001_R1_001.fastq.gz | 62112 | 40037 | 39598 | 37825 |
| 087-035_^CCATGCGTC_L001_R1_001.fastq.gz | 1 | 1 | 1 | 1 |
| 087-040_^ACATGGCTC_L001_R1_001.fastq.gz | 27755 | 22602 | 22049 | 21806 |
| 087-041_^ACATGTAGG_L001_R1_001.fastq.gz | 32098 | 25486 | 24954 | 24726 |
| 087-042_^TAGAGGATC_L001_R1_001.fastq.gz | 31155 | 24385 | 23941 | 23690 |
| 087-043_^ACGATCACT_L001_R1_001.fastq.gz | 33942 | 27618 | 27135 | 26851 |
| 087-044_^GCGATGTGG_L001_R1_001.fastq.gz | 29618 | 23681 | 23167 | 22916 |
| 087-045_^CTACGCATC_L001_R1_001.fastq.gz | 14344 | 10891 | 10652 | 10546 |
| 087-046_^TCGCGCATC_L001_R1_001.fastq.gz | 29786 | 22937 | 22619 | 22290 |
| 087-047_^TATATCACT_L001_R1_001.fastq.gz | 19119 | 15320 | 14927 | 14881 |
| 087-601_^ATATAGTTC_L001_R1_001.fastq.gz | 27596 | 22943 | 22610 | 22515 |
| 087-602_^GACCACGCT_L001_R1_001.fastq.gz | 37526 | 31615 | 31277 | 30881 |
| 087-603_^CTCAACTCT_L001_R1_001.fastq.gz | 27893 | 20649 | 20288 | 20158 |
| 087-604_^GCCGAGATC_L001_R1_001.fastq.gz | 18734 | 14281 | 13977 | 13936 |
| 087-605_^ACCGAGACT_L001_R1_001.fastq.gz | 45910 | 39040 | 38637 | 37955 |
| 087-606_^CACCAGCTC_L001_R1_001.fastq.gz | 35460 | 28175 | 27783 | 27743 |
| 087-609_^GACCATATC_L001_R1_001.fastq.gz | 36039 | 28828 | 28428 | 26958 |
| 087-610_^CTCAATCTC_L001_R1_001.fastq.gz | 21824 | 17069 | 16812 | 16782 |
| 087-612_^GGCTATGCT_L001_R1_001.fastq.gz | 34168 | 28705 | 28462 | 28278 |
| 087-613_^GACACATTC_L001_R1_001.fastq.gz | 33400 | 27133 | 26806 | 26674 |
| 087-614_^CACACATGG_L001_R1_001.fastq.gz | 43476 | 34285 | 33949 | 32518 |
| 087-615_^TACACCGTC_L001_R1_001.fastq.gz | 28979 | 22803 | 22518 | 21958 |
| 087-617_^GCCAGAGTC_L001_R1_001.fastq.gz | 37326 | 26922 | 26472 | 26360 |
| 087-618_^CACTGATTC_L001_R1_001.fastq.gz | 39726 | 30741 | 30445 | 29514 |
| 087-619_^GACTGCGTC_L001_R1_001.fastq.gz | 37864 | 31731 | 31459 | 30059 |
| 087-620_^TGCATGTGG_L001_R1_001.fastq.gz | 38379 | 27475 | 27182 | 25921 |
| 087-621_^CACGTTACT_L001_R1_001.fastq.gz | 32415 | 26622 | 26250 | 25566 |
| 087-622_^TGCATTGCT_L001_R1_001.fastq.gz | 19496 | 15794 | 15539 | 15375 |
| 087-623_^ATGCAATCT_L001_R1_001.fastq.gz | 51577 | 39810 | 39457 | 39015 |
| 087-624_^TTGCACGTC_L001_R1_001.fastq.gz | 17738 | 14314 | 14101 | 13695 |
| 087-625_^CGTACCGTC_L001_R1_001.fastq.gz | 14635 | 12015 | 11798 | 11798 |
| 087-626_^CATCGCAGG_L001_R1_001.fastq.gz | 21691 | 16796 | 16577 | 16304 |
| 087-627_^TATATACGG_L001_R1_001.fastq.gz | 16843 | 13965 | 13741 | 13606 |
| 087-628_^CTGACGCCT_L001_R1_001.fastq.gz | 21437 | 17424 | 17062 | 16902 |
| 087-630_^CTGCACGCT_L001_R1_001.fastq.gz | 28386 | 20341 | 20034 | 20020 |
| 087-632_^CCGCGTGTC_L001_R1_001.fastq.gz | 21839 | 17831 | 17519 | 17469 |
| 087-633_^CCGCGCACT_L001_R1_001.fastq.gz | 25366 | 19614 | 19353 | 18816 |
| 087-634_^GCGCGCAGG_L001_R1_001.fastq.gz | 18003 | 14158 | 13922 | 13889 |
| 087-640_^ATGACCGCT_L001_R1_001.fastq.gz | 35344 | 29837 | 29477 | 29357 |
| 087-con2_^ATGGTCGTC_L001_R1_001.fastq.gz | 1 | 1 | 1 | 1 |
| 087-con3_^GTGGTCGCT_L001_R1_001.fastq.gz | 2 | 2 | 2 | 2 |
| 087-con4_^TATTAGTCT_L001_R1_001.fastq.gz | 1 | 1 | 1 | 1 |
| 087-con5_^TCTAATCTC_L001_R1_001.fastq.gz | 298 | 248 | 213 | 213 |
| 087-con6_^CGTTGATCT_L001_R1_001.fastq.gz | 5 | 3 | 1 | 1 |
| neg16_^TCAGTGCTC_L001_R1_001.fastq.gz | 23 | 9 | 6 | 6 |
| neg3_^GTATAGTCT_L001_R1_001.fastq.gz | 22 | 10 | 9 | 9 |
| neg8_^ATACGACTC_L001_R1_001.fastq.gz | 13 | 5 | 2 | 2 |
| Pos1_^CGTGTTATC_L001_R1_001.fastq.gz | 23186 | 19416 | 19362 | 19362 |
| Pos2_^ATTGAGATC_L001_R1_001.fastq.gz | 24790 | 21161 | 21103 | 21103 |
| Pos3_^TCACACGCT_L001_R1_001.fastq.gz | 26357 | 22141 | 22087 | 22087 |
